# Three decades of recombinant human growth hormone research in children born small for gestational age: a bibliometric analysis (1996–2025)

**DOI:** 10.3389/fped.2026.1903046

**Published:** 2026-09-09

**Authors:** Gülin Karacan Küçükali, Hasan Bora Ulukapı, Burçe Orman, Şenay Savaş Erdeve

**Affiliations:** 1 Department of Pediatric Endocrinology, Ankara Etlik City Hospital, Ankara, Türkiye; 2 Department of Pediatric Endocrinology, University of Health Sciences, Ankara, Türkiye

**Keywords:** BERTopic, bibliometric analysis, growth hormone, scientometrics, SGA, small for gestational age

## Abstract

**Background:**

Recombinant human growth hormone (rhGH) has been a standard therapy for short children born small for gestational age (SGA) since 2001 (FDA) and 2003 (EMA), yet no bibliometric analysis has mapped this field's intellectual structure.

**Materials and methods:**

Publications were retrieved from four databases, Lens.org (*n* = 1,184), OpenAlex (*n* = 1,343), Web of Science Core Collection (*n* = 1,121), and Scopus (*n* = 1,589), yielding 793 unique records after DOI-based merging, therapeutic-relevance filtering, conference-abstract removal, and expert-led relevance auditing, restricted to the 30 complete calendar years 1996–2025. Analyses included publication trends, Bradford's and Lotka's Laws, co-authorship and keyword co-occurrence networks, geographic mapping, citation metrics, open access (OA) trends, keyword trend analysis across three regulatory phases, and transformer-based topic modeling (BERTopic).

**Results:**

The field showed three phases temporally aligned with regulatory milestones, stabilizing at a compound annual growth rate (CAGR) of 6.3% (2011–2025). Hormone Research in Paediatrics (*n* = 107) and the Journal of Clinical Endocrinology & Metabolism (*n* = 89) were the leading sources. Hokken-Koelega ACS was the most prolific author (*n* = 81, combining name variants). The United States (*n* = 115), the Netherlands (*n* = 111), and Germany (*n* = 95) led in output across 67 countries. The corpus-level h-index was 66, with 19,415 total citations and a Gini coefficient of 0.69 indicating substantial citation inequality. An unadjusted comparison of citations by open-access status was null (*p* = 0.86); however, after adjusting for citation age and document type, open-access articles received 2.2-fold more citations than closed-access articles (incidence-rate ratio 2.20, 95% CI 1.84–2.63), an association that attenuated but persisted within journals (1.45). BERTopic identified 7 research themes from 771 abstracts, showing a temporal shift from basic efficacy studies toward molecular and genetic diagnostics (GH–IGF-I axis, Silver–Russell syndrome, genetic short stature) and patient-centered outcomes. Keyword trend analysis across three regulatory phases identified 8 terminological shifts that remained significant after false-discovery-rate correction, including a decline in “IUGR” usage (*χ*^2^ = 66.9, *p* < 0.001) and a rise in observational/registry studies (*χ*^2^ = 37.6, *p* < 0.001).

**Conclusion:**

The rhGH–SGA literature shows a maturing research landscape with increasing thematic diversification from efficacy studies toward molecular and genetic diagnostics and patient-centered outcomes. Keyword trend analysis reveals measurable shifts in terminology, study methodology, and patient populations, identifying gaps and emerging directions that can inform future trial design and funding priorities.

## Introduction

Children born small for gestational age (SGA), defined as birth weight and/or length below −2 standard deviations (SD) for gestational age, represent 3%–10% of all live births globally (1). While most SGA infants demonstrate spontaneous catch-up growth within the first 2 years of life, 10%–15% remain short, with adult heights substantially below the normal range if untreated (2, 3). Recombinant human growth hormone (rhGH) therapy is the primary pharmacological intervention for these children, with regulatory approval by the U.S. Food and Drug Administration (FDA) in 2001 and the European Medicines Agency (EMA) in 2003 (3).

Since the early clinical trials of the mid-1990s (4), the evidence base for rhGH treatment in SGA has grown to encompass randomized controlled trials (5, 6), long-term safety surveillance (7, 8), metabolic outcome studies (9), and molecular investigations into growth hormone axis physiology and treatment sensitivity (10). Key consensus statements (1–3) have periodically synthesized this evidence. Why, then, should this field also be examined bibliometrically? Because a consensus statement reports what the evidence shows, not how the evidence base itself is built: which groups and journals generate it, how international it has become, and how its central questions have shifted across three decades of regulatory change. Those structural features determine where the field's momentum and its gaps lie, and they are not visible from narrative synthesis alone.

Bibliometric analysis answers exactly these questions, quantifying publication trends, collaboration networks, and thematic evolution within a research domain (11). While recent bibliometric studies have examined idiopathic short stature (12, 13), short stature broadly (14), and intrauterine growth restriction (15), no analysis has specifically addressed GH axis research and rhGH treatment in the SGA population—a clinically and biologically distinct entity with its own regulatory pathway, treatment criteria, and outcome measures.

This gap is methodologically relevant. The SGA–GH field has evolved from fundamental efficacy questions (“Does GH increase adult height?”) toward precision medicine challenges: pharmacogenomic predictors of treatment response (e.g., d3-GHR polymorphism), long-acting GH formulations [somapacitan (16), lonapegsomatropin (17)], cardiometabolic risk profiling, and epigenetic diagnostics for Silver–Russell syndrome. Mapping this thematic trajectory can guide clinicians and funders toward the most productive research frontiers. Moreover, the literature straddles multiple specialties—pediatric endocrinology, neonatology, obstetrics, genetics, and pharmacology—making it difficult for any single researcher to grasp the full scope without a systematic bibliometric overview.

We therefore present, to our knowledge, the first bibliometric analysis of recombinant human growth hormone research in SGA children, covering 793 publications from 1996 to 2025. Four-database retrieval is combined with classical bibliometric laws, co-authorship and keyword networks, keyword trend analysis across three regulatory phases, and transformer-based topic modeling with BERTopic (18), so that structural and semantic perspectives complement one another.

## Materials and methods

**Figure 1:**
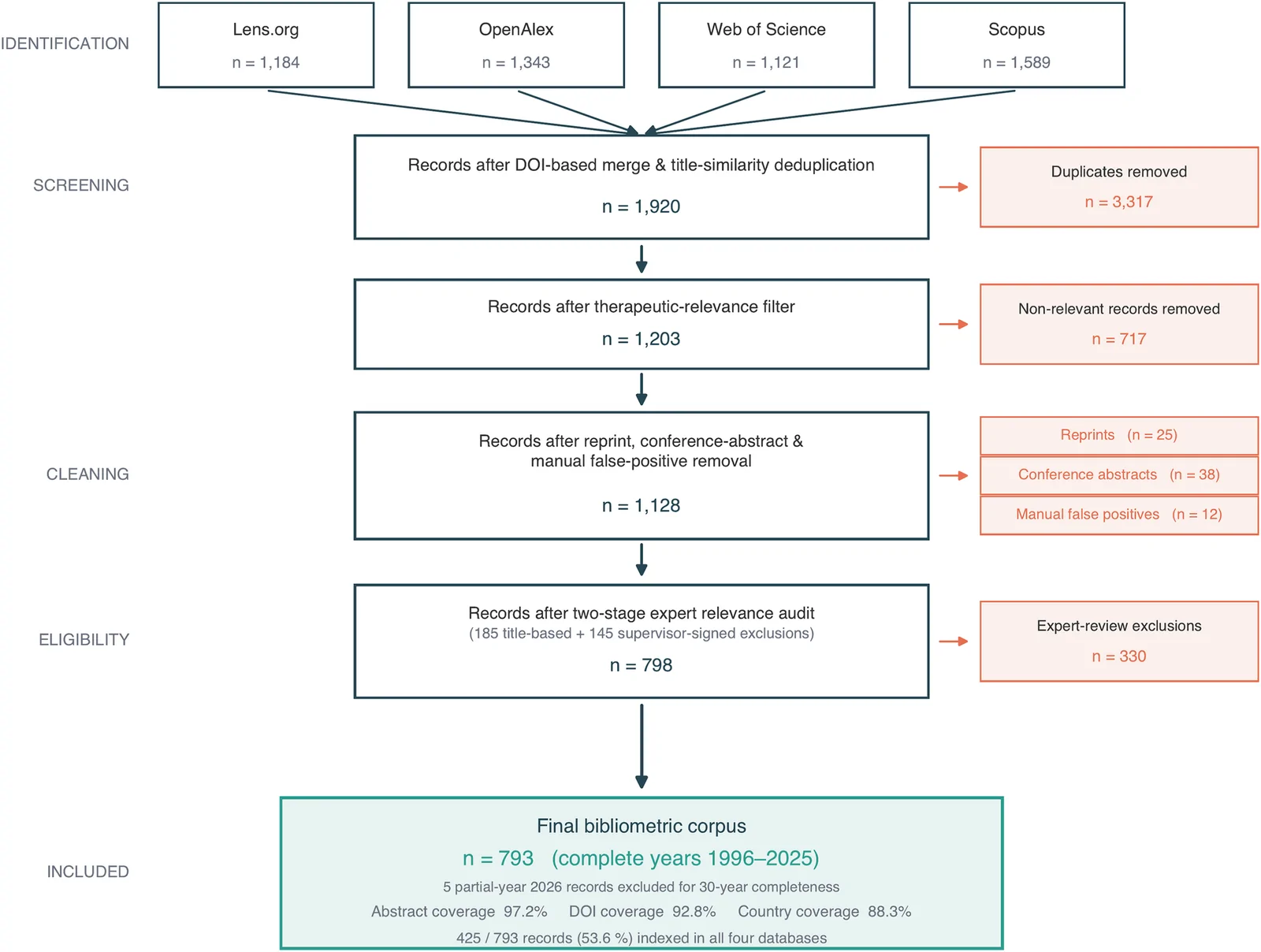
PRISMA-style flowchart of the study selection process.

This study used a bibliometric design to map the intellectual structure and thematic evolution of GH axis research and rhGH treatment in children born SGA, 1996–2025 (Figure 1). The protocol followed the framework of Donthu et al. (11) and the STAR Protocol (19) and was reported in line with the BIBLIO checklist (20) and the GLOBAL recommendations (21).

### Data sources and search strategy

Four databases; Lens.org and OpenAlex, and Web of Science (WoS) Core Collection and Scopus were searched in February 2026. Boolean queries combining the same two concept sets were applied across databases, with syntactic adaptations for each query language (TS = for WoS, TITLE-ABS-KEY for Scopus). The search combined SGA-related terms (“small for gestational age,” “SGA,” “intrauterine growth retardation,” “IUGR,” “fetal growth restriction”) with GH-treatment terms (“growth hormone,” “somatotropin,” “rhGH,” “GH therapy”); the OpenAlex query additionally included “intrauterine growth restriction,” “born SGA,” “somatropin,” and “GH treatment,” and all searches were restricted to journal articles and reviews published in 1996–2025. Initial yields were 1,184 (Lens.org), 1,343 (OpenAlex), 1,121 (WoS), and 1,589 (Scopus) records. The complete query strings for every database are provided in Supplementary Text S1.

### Screening and deduplication

Records were merged across databases by normalized DOI; records lacking a DOI were matched by 80-character title-prefix comparison. Screening was two-stage. An automated therapeutic-relevance filter retained records whose title, abstract, keywords, or MeSH terms contained both an SGA-related term and a direct GH-treatment or auxological-outcome term, excluding peripheral IGF-biology, placental, and animal-model papers. The retained records then underwent expert relevance auditing by the study team with clinician sign-off, which removed a further set of off-topic or duplicate records; because this stage was a consensus audit rather than two independent blinded ratings, an inter-rater kappa was not computed, and this is noted as a limitation. Conference abstracts and reprints were removed by DOI pattern. The final corpus comprised 793 publications; 425 (53.6%) were indexed in all four databases (Figure 1).

### Descriptive and trend analysis

Annual publication counts were stratified into three previously-defined regulatory phases: Phase 1/Pre-Approval Era (1996–2000), Phase 2/Approval & Guidelines Era (2001–2010), and Phase 3/Maturation & Modern Era (2011–2025), with compound annual growth rates (CAGR) computed for each phase on cumulative counts. Because the search was run in early 2026, a small number of 2026-dated records were retrieved; to preserve a complete 30-year window (1996–2025), these five records were excluded from all analyses. Key milestones—FDA approval (2001), EMA approval (2003) (3), the Clayton (2007) (1) and Hokken-Koelega (2023) (2) consensus statements—were annotated on trend figures.

### Classical bibliometric laws

Bradford's Law of scattering identified core journals by ranking journals in descending productivity and dividing them into three zones each containing approximately one-third of total publications. Lotka's Law was assessed by fitting an inverse power law to the author-publication frequency distribution, with goodness-of-fit evaluated by the Kolmogorov–Smirnov test.

### Network analysis

Co-authorship networks were constructed from author pairs within each publication, restricted to authors with ≥3 publications. Keyword co-occurrence networks were built from OpenAlex concept tags (92.4% coverage); 37 high-level generic concept terms (e.g., “Medicine,” “Biology”) were removed and the top 80 domain-specific keywords retained, with edges filtered at ≥10 co-occurrences. Community structure was detected with the Louvain algorithm (22) using community-seeded spring-force layouts. Analyses were implemented in NetworkX (Python).

### Geographic and citation analyses

Country and institutional productivity were derived from OpenAlex affiliation data (88.3% coverage), with international collaboration assessed using single-country (SCP) versus multiple-country (MCP) publication counts. Citation metrics included the corpus-level h-index (23), g-index (24), the Gini coefficient of citation inequality, and the proportion of uncited papers. Citation bursts were identified using a sliding-window Z-score method on citation-to-year-average ratios.

### Open access analysis

OA status was classified as gold, green, bronze, hybrid, or closed using OpenAlex metadata. Because citation counts depend on how long a paper has been in circulation and on its document type, the association between OA status and citations was estimated primarily with a negative-binomial regression of citation count on OA status, adjusting for citation age (log years since publication) and document type (original article vs. review); document type was recovered by DOI from the four source databases (92% coverage). The unadjusted Mann–Whitney U test (with rank-biserial and Cohen's d effect sizes) and an age-normalised comparison of citations per year are reported alongside the adjusted model. Robustness was assessed by excluding reviews, adding publication-year and journal fixed effects, excluding the most-cited papers, restricting OA to gold/hybrid routes, and stratifying by publication era.

### Keyword trend analysis

Terminological evolution was quantified by tracking keyword frequencies in titles and abstracts across the three regulatory phases defined above (Phase 1, *n* = 65; Phase 2, *n* = 242; Phase 3, *n* = 486). Eight *a priori* thematic dimensions (GH treatment approaches, dosing strategies, clinical outcomes, safety/metabolic parameters, GH responsiveness/indication, patient populations, study methodologies, and guideline/regulatory terms) were analyzed, with cross-phase differences tested by chi-square tests of independence. Because 37 tests were performed, the Benjamini–Hochberg false-discovery-rate correction was applied across the full family of tests; trends are reported as significant only when they survived correction at q < 0.05 (Bonferroni-corrected results were identical).

### Topic modeling with BERTopic

Latent thematic structure was identified with BERTopic (18), which combines pretrained sentence embeddings with density-based clustering. Abstracts were embedded with all-MiniLM-L6-v2, a compact Sentence-BERT sentence-embedding model (25) (not a generative large language model), projected to a low-dimensional space via UMAP (26), and clustered with HDBSCAN (27), which assigns thematically ambiguous abstracts to an outlier class rather than forcing them into the nearest cluster. Of 793 articles, 771 (97.2%) had abstracts available; residual structured-abstract markup was stripped before embedding. BERTopic was chosen over latent Dirichlet allocation (LDA) and non-negative matrix factorisation (NMF) for its explicit outlier handling, its use of transfer-learned semantic embeddings (advantageous for a corpus of this size and for a field whose terminology shifted over three decades), and the interpretability of its topics; on this corpus its mean topic coherence (NPMI 0.15) was comparable to LDA (0.14) and NMF (0.18) (Supplementary Text S2). Clustering used min_cluster_size = 10 and min_samples = 3, a configuration that recovered the same seven-topic solution on all ten random seeds tested; a 20-configuration sensitivity analysis (Supplementary Figure S1) and full hyperparameters and software versions are reported in the Supplementary Material. The selected solution produced 7 interpretable topics with a 21.5% outlier rate (*n* = 166 abstracts).

### Software and statistical analysis

Analyses were conducted in Python 3.14.3 (pandas, NumPy, SciPy, matplotlib, NetworkX, python-louvain, BERTopic 0.17.4, sentence-transformers, UMAP-learn, scikit-learn) and R 4.4.1 (bibliometrix 5.2.1 (28), ggplot2, tidyverse, ggraph). Statistical significance was set at *p* < 0.05.

### Ethics statement

This study analyzed publicly available, anonymized bibliographic metadata and did not involve human participants, identifiable patient data, or animal subjects; institutional review board approval and informed consent were therefore not required.

## Results

### Data retrieval and screening

The four-database search yielded a final corpus of 793 publications spanning the 30 complete calendar years 1996–2025 (Figure 1). Of these, 425 records (53.6%) were indexed in all four databases. Database coverage was: Lens.org 655 (82.6%), Scopus 646 (81.5%), OpenAlex 637 (80.3%), and WoS 567 (71.5%). The merged dataset had 97.2% abstract coverage, 92.8% DOI coverage, and 88.3% country-affiliation coverage.

### Publication trends

**Figure 2:**
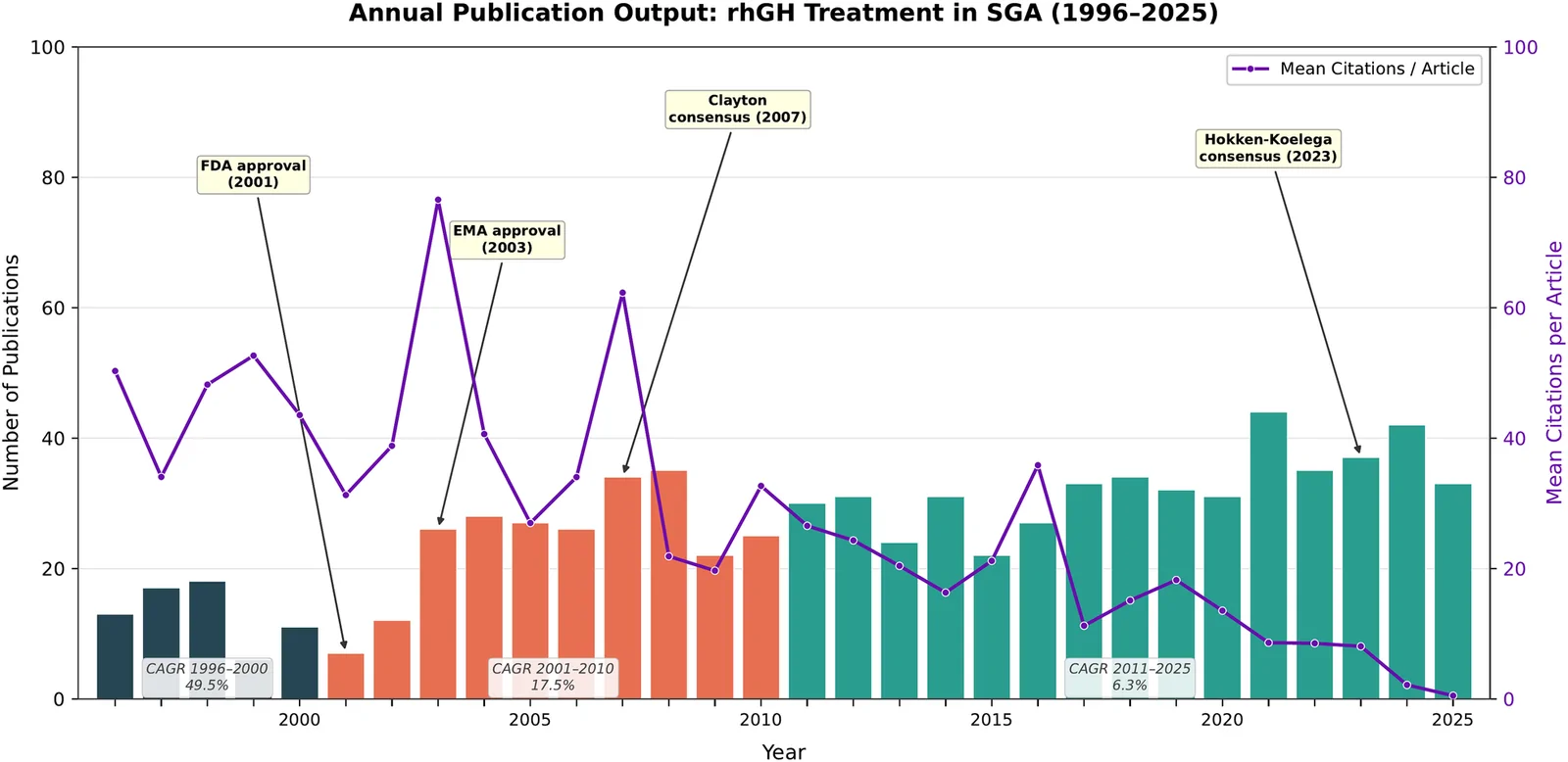
Annual publication output and mean citations per article, 1996–2025, color-coded by regulatory phase (phase 1, 1996–2000; phase 2, 2001–2010; phase 3, 2011–2025), with per-phase CAGR and key regulatory and consensus milestones annotated. Year-by-year data are in Supplementary Table S1.

Figure 2 presents the annual publication output from 1996 to 2025. The field exhibited three distinct regulatory phases:

1. Phase 1 — Pre-Approval Era (1996–2000): CAGR = 49.5%. Annual output rose rapidly during this period, temporally aligned with the first clinical trials of rhGH in SGA (4).
2. Phase 2 — Approval & Guidelines Era (2001–2010): CAGR = 17.5%. Publication counts increased following FDA approval (2001) and EMA approval (2003), peaking at 35 publications in 2008. The Clayton consensus statement (1) preceded a further rise in output.
3. Phase 3 — Maturation & Modern Era (2011–2025): CAGR = 6.3%. Annual output stabilized at approximately 22–44 publications per year, peaking at 44 in 2021.

### Journal analysis

Table 1 presents the top 15 journals by publication count. Hormone Research in Paediatrics (including its predecessor Hormone Research, merged as the same journal) was the dominant venue (*n* = 107, 13.5%), followed by the Journal of Clinical Endocrinology & Metabolism (JCEM; *n* = 89, 11.2%).

**Table 1:** Top 15 journals by publication count in rhGH–SGA research (1996–2025).

Rank	Journal	*n*	%
1	Horm Res Paediatr^a^	107	13.5
2	J Clin Endocrinol Metab	89	11.2
3	J Pediatr Endocrinol Metab	44	5.5
4	Clin Endocrinol	32	4.0
5	Eur J Endocrinol	25	3.2
6	Front Endocrinol	17	2.1
7	Growth Horm IGF Res	16	2.0
8	Clin Pediatr Endocrinol	13	1.6
9	Acta Paediatr	12	1.5
10	Int J Pediatr Endocrinol	11	1.4
11	J Endocrinol Invest	10	1.3
12	Pediatr Endocrinol Rev	10	1.3
13	Pediatrics	10	1.3
14	J Clin Res Pediatr Endocrinol	9	1.1
15	Best Pract Res Clin Endocrinol Metab	8	1.0

a Includes predecessor title *Hormone Research* [1996–2009]; journal renamed in 2010.

**Figure 3:**
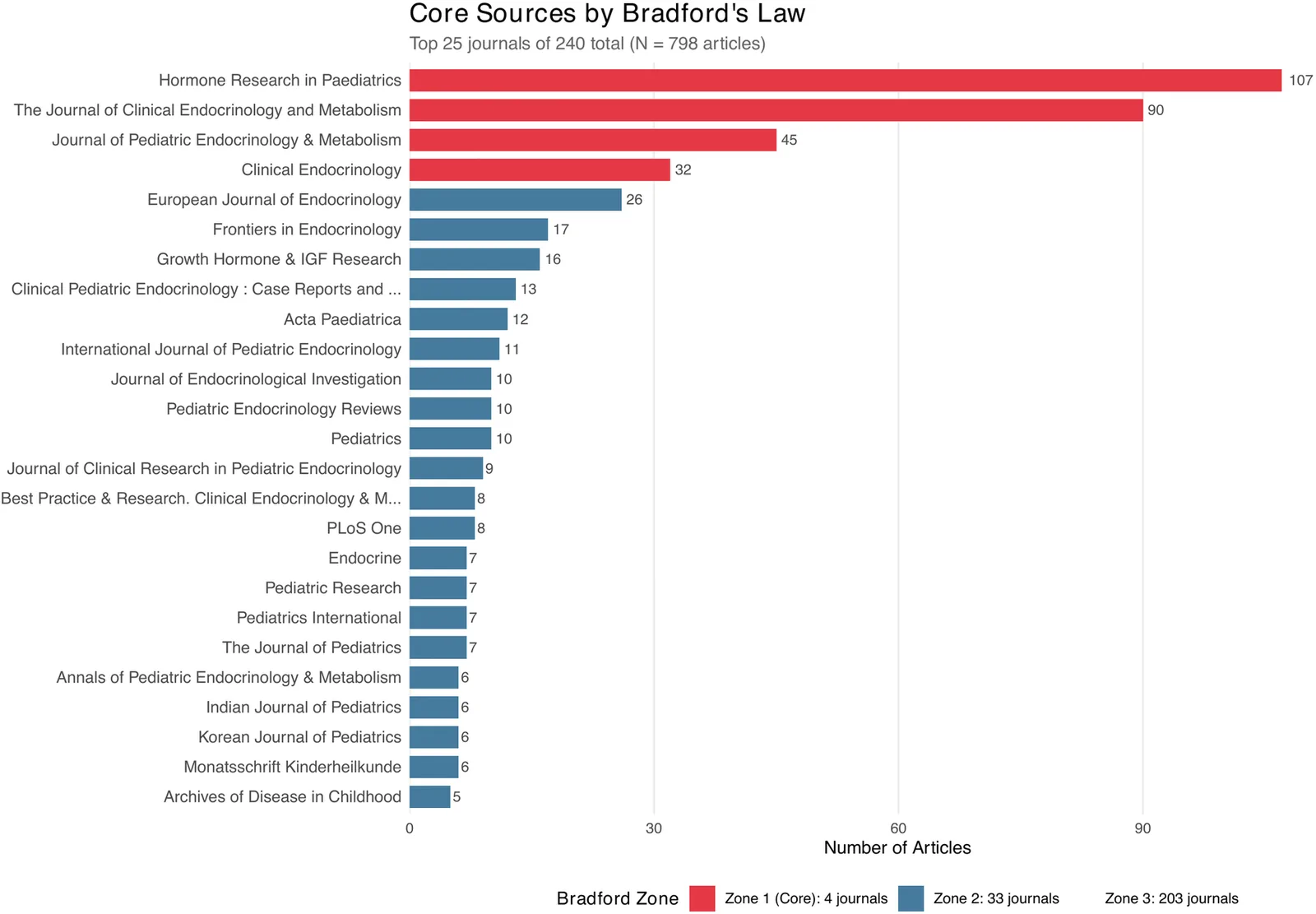
Bradford's Law of scattering: journals divided into three productivity zones. Zone 1 (core) journals produce approximately one-third of all publications (Journals out of top 25 within zones 2 and 3 are not shown in this chart).

Bradford's Law analysis (Figure 3) divided journals into three zones, with Zone 1 (core) containing 4 journals (of 240 total), Zone 2 containing 34 journals, and Zone 3 containing the remaining 202 journals.

### Author productivity

The 793 publications involved a large author community (*n* = 2,985 unique authors). Table 2 shows the top 15 most productive authors. Hokken-Koelega ACS (Erasmus MC, Netherlands) was the most prolific contributor with 81 publications (combining name variants), followed by Ranke MB (University of Tübingen, Germany; *n* = 22) and Albertsson-Wikland K (University of Gothenburg, Sweden; *n* = 18).

**Table 2:** Top 15 most productive authors in rhGH–SGA research.

Rank	Author	*n*	GCS	Primary Affiliation
1	Hokken-Koelega, ACS	81	4,671	Erasmus MC, Netherlands
2	Ranke, MB	22	1,105	Univ. Tübingen, Germany
3	Albertsson-Wikland, K	18	1,159	Univ. Gothenburg, Sweden
4	de Zegher, F	17	846	KU Leuven, Belgium
5	van der Steen, M	17	601	Erasmus MC, Netherlands
6	Tanaka, T	16	329	Natl. Center Child Health, Japan
7	Dunger, DB	15	426	Univ. Cambridge, UK
8	Juul, A	14	302	Univ. Copenhagen, Denmark
9	Wit, JM	14	727	Leiden Univ., Netherlands
10	Mulder, PGH	14	1,254	Erasmus MC, Netherlands
11	Lindberg, A	13	722	Pfizer, Sweden
12	Willemsen, RH	13	339	Erasmus MC, Netherlands
13	Yokoya, S	11	164	Natl. Center Child Health, Japan
14	Cianfarani, S	11	1,018	Bambino Gesù Hospital, Italy
15	Lee, PA	11	893	Penn State, USA

GCS, global citation score. Name variants were merged for the most prolific authors (Hokken-Koelega, Albertsson-Wikland, de Zegher, Cianfarani).

Lotka's Law analysis (Supplementary Figure S2) showed an inverse power distribution (*β* = 2.25, R^2^ = 0.92, Kolmogorov–Smirnov *p* = 0.300), steeper than Lotka's classical exponent of 2.0, indicating more concentrated productivity than the theoretical prediction.

### Co-authorship network

**Figure 4:**
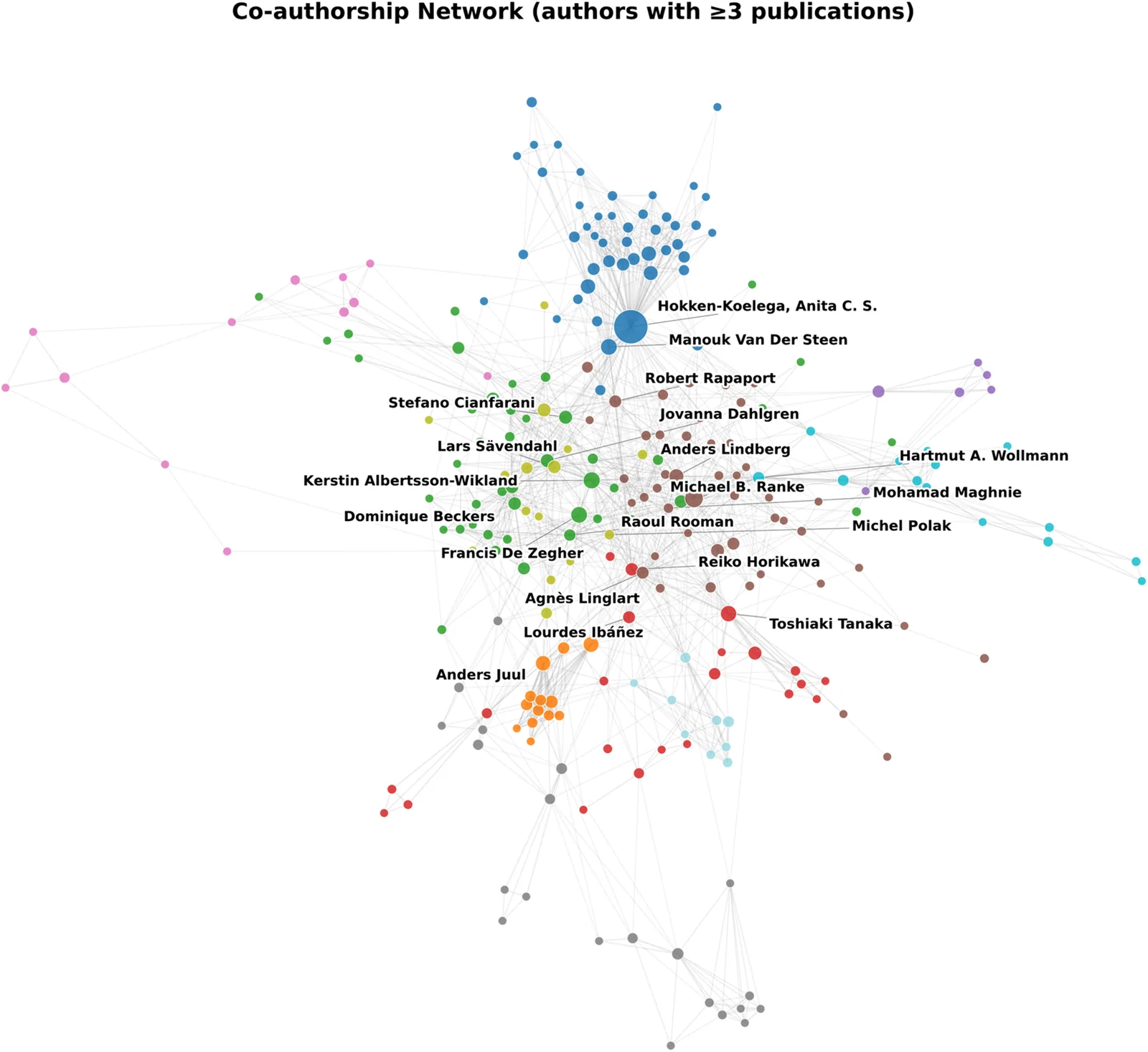
Co-authorship network of researchers with ≥3 publications. Node size reflects publication count; colors indicate Louvain communities.

The co-authorship network of researchers with ≥3 publications (Figure 4; largest connected component, 252 nodes and 1,410 edges) resolved into several densely connected clusters: a large European group centered on Anita Hokken-Koelega (Netherlands), linked to Jan-Maarten Wit and Manouk van der Steen (Netherlands) and Kerstin Albertsson-Wikland (Sweden); a German–Swiss group around Michael Ranke and Gerhard Binder; a Scandinavian group linking Anders Juul and Rikke Beck Jensen (Denmark); and a less internationally connected Asia–Pacific group (Reiko Horikawa, Toshiaki Tanaka, Japan). This structure mirrors the field's development through major multi-center trials and consensus collaborations.

### Keyword co-occurrence analysis

**Figure 5:**
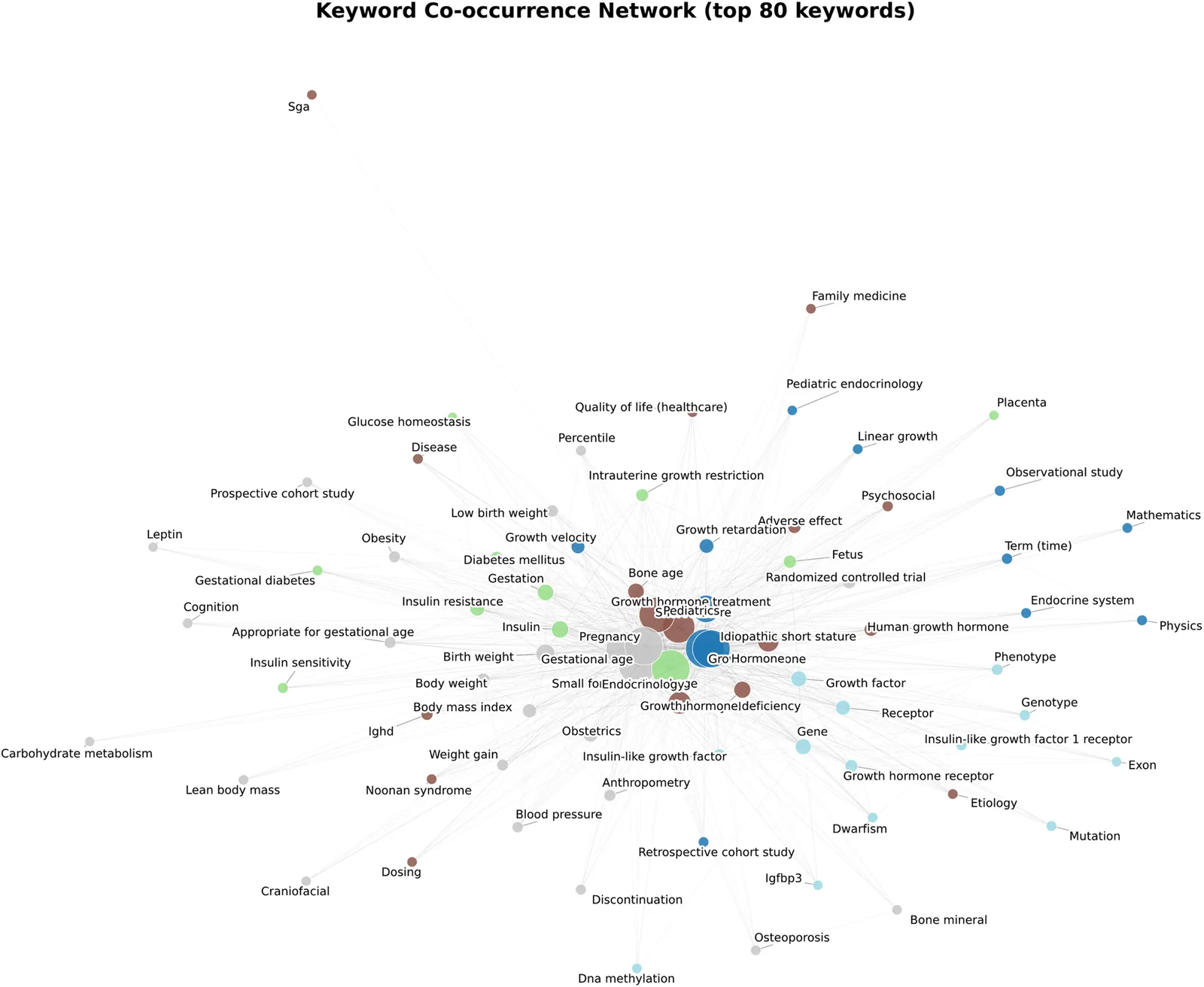
Keyword co-occurrence network (top 80 keywords). Node size reflects frequency; colors indicate thematic communities detected by Louvain algorithm.

After removing 37 generic disciplinary labels from the OpenAlex concept tags (see Methods), the keyword co-occurrence network (Figure 5) comprised 78 nodes and 743 edges, resolving into five thematic clusters via Louvain community detection: an obstetric/perinatal cluster (“small for gestational age,” “birth weight,” “gestational age”); a clinical-diagnoses cluster (“short stature,” “growth hormone deficiency,” “Turner syndrome”); a molecular-endocrinology cluster (“insulin-like growth factor,” “receptor,” “insulin resistance”); a GH-therapeutics cluster (“growth hormone treatment,” “growth velocity,” “randomized controlled trial”); and a genetics/imprinting cluster (“epigenetics,” “methylation,” “Silver–Russell syndrome”).

A thematic map (strategic diagram, Supplementary Figure S3) was generated using keyword clustering, positioning themes along centrality (relevance to the field) and density (internal coherence) axes. Motor themes (high centrality, high density) included GH treatment efficacy and adult height, while emerging/declining themes were mapped to the respective quadrants.

### Geographic distribution

**Figure 6:**
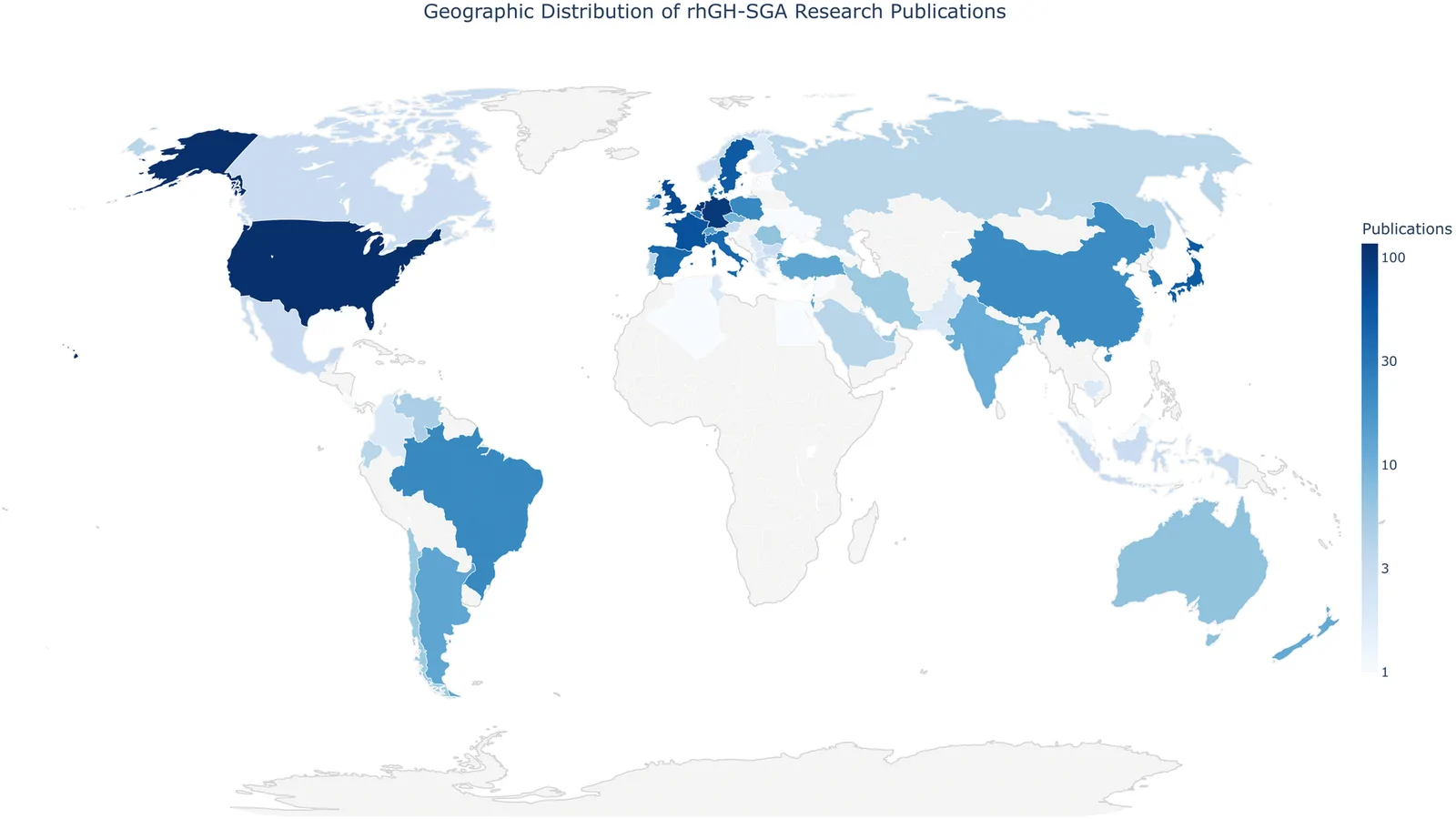
World choropleth map showing geographic distribution of rhGH–SGA research publications.

Geographic analysis, based on OpenAlex affiliation data available for 88.3% of the corpus spanning 67 countries, showed a Western European and North American concentration (Figure 6). The United States led in absolute publication count (*n* = 115, 14.5%), followed by the Netherlands (*n* = 111, 14.0%), Germany (*n* = 95, 12.0%), the United Kingdom (*n* = 73, 9.2%), and France (*n* = 64, 8.1%).

International collaboration was present in 52.2% of records with country data (MCP ratio), reflecting both independent national programs and coordinated multi-center trials. The top 10 contributing countries with collaboration metrics are presented in Supplementary Table S2.

### Citation analysis

**Figure 7:**
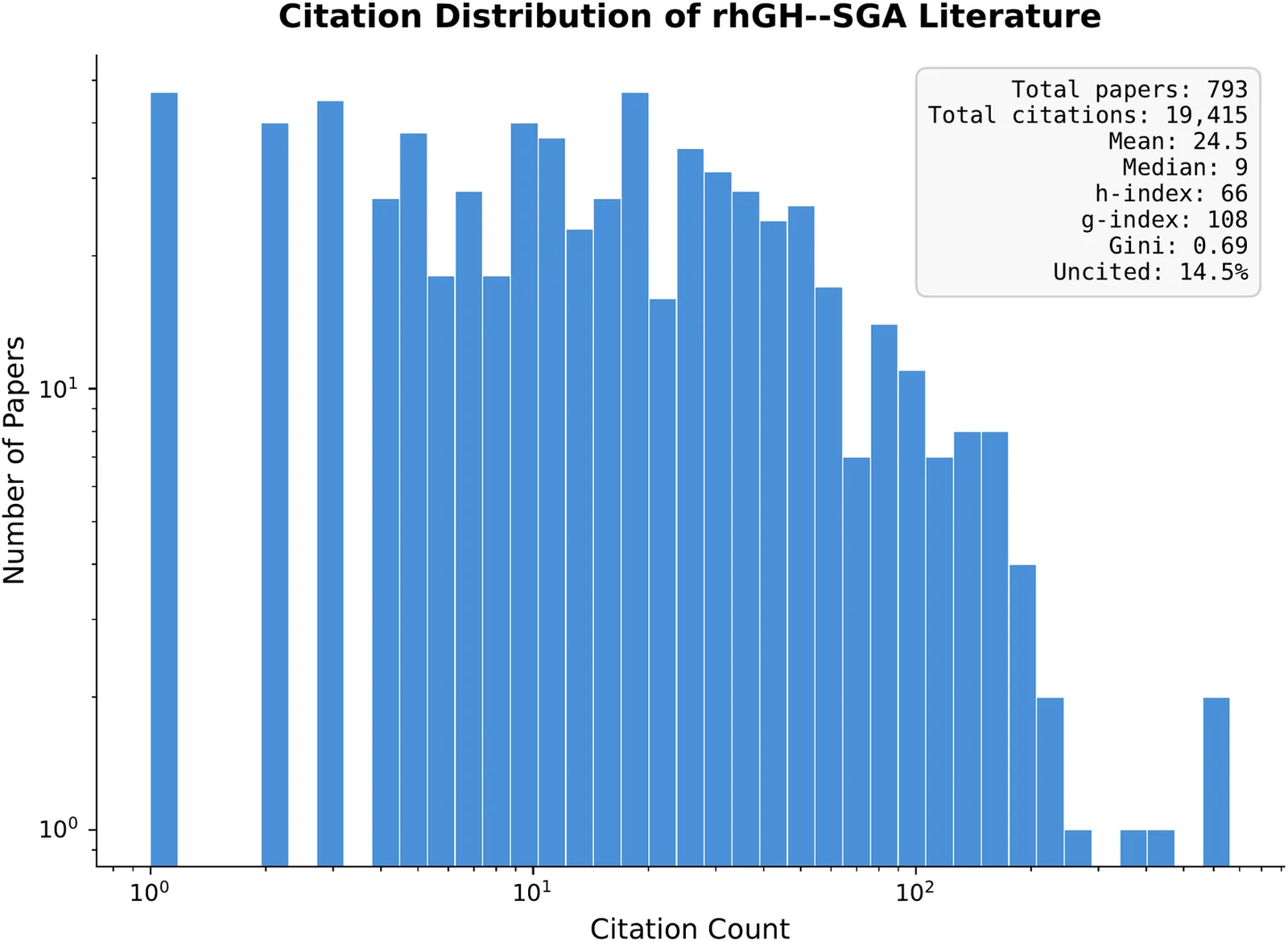
Citation distribution of the rhGH–SGA literature (log–log scale). The right-skewed distribution and high Gini coefficient (0.69) indicate substantial citation inequality.

The corpus accumulated 19,415 total citations, with a mean of 24.5 and a median of 9.0 citations per paper. The h-index was 66 and the g-index was 108. The Gini coefficient of 0.69 indicated substantial citation inequality—a small number of landmark papers attracted the majority of citations—while 14.5% of papers remained uncited (Figure 7). Excluding the most-cited papers confirmed that this inequality is a structural feature of the field rather than an artifact of a few landmark publications (Gini excl. top 5 = 0.657, excl. top 10 = 0.647).

The most cited paper was the Clayton et al. (2007) consensus statement (1) (662 citations), followed by the Lee et al. (2003) consensus (3) (589 citations), the Wakeling et al. (2017) Silver–Russell syndrome consensus (29) (438 citations), and the Saenger et al. (2007) comprehensive review (30) (390 citations). The top-cited papers were predominantly review or consensus articles rather than original research, reflecting the field's reliance on authoritative guidance documents as reference anchors. Table 3 presents the 15 most cited articles in the corpus.

**Table 3:** Fifteen most cited articles in rhGH–SGA research.

Article	Year	GCS	DOI
Clayton PE et al. Management of the child born SGA. J Clin Endocrinol Metab.	2007	662	10.1210/jc.2006-2017
Lee PA et al. International SGA Advisory Board consensus. Pediatrics.	2003	589	10.1542/peds.111.6.1253
Wakeling E et al. Silver–Russell syndrome: first international consensus. Nat Rev Endocrinol.	2017	438	10.1038/nrendo.2016.138
Saenger P et al. Small for gestational age: short stature and beyond. Endocr Rev.	2007	390	10.1210/er.2006-0039
van Pareren Y et al. Adult height after long-term GH treatment in SGA…J Clin Endocrinol Metab.	2003	270	10.1210/jc.2002-021172
Albertsson-Wikland K et al. SGA: postnatal growth and hormonal status. Horm Res Paediatr.	1998	209	10.1159/000053080
Takeda A et al. rhGH for growth disorders in children: HTA review. Health Technol Assess.	2010	207	10.3310/hta14420
Sas TCJ et al. GH treatment in short children born SGA: 5-year dose-response trial. J Clin Endocrinol Metab.	1999	205	10.1210/jcem.84.9.5942
Ranke MB et al. Prediction of GH response in short SGA children: KIGS data. J Clin Endocrinol Metab.	2003	183	10.1203/01.PDR.0000148716.71231.81
Dahlgren J, Albertsson-Wikland K. Final height in SGA children treated with GH. Pediatr Res.	2005	183	10.1203/01.PDR.0000148716.71231.81
Finken MJJ et al. Children born SGA: molecular genetics and implications. Endocr Rev.	2018	176	10.1210/er.2018-00083
Léger J et al. Growth factors and IUGR…Pediatr Res.	1996	171	10.1203/00006450-199607000-00018
Sävendahl L et al. Long-term mortality in GHD/ISS/SGA treated with rhGH (SAGhE). J Clin Endocrinol Metab.	2012	171	10.1210/jc.2011-2882
Binder G et al. The d3-GHR polymorphism & GH responsiveness in Turner and SGA. J Clin Endocrinol Metab.	2005	167	10.1210/jc.2005-1581
Gluckman PD, Harding JE. Physiology and pathophysiology of IUGR. Horm Res Paediatr.	1997	166	10.1159/000191257

GCS, global citation score. Article titles abbreviated for space.

**Figure 8:**
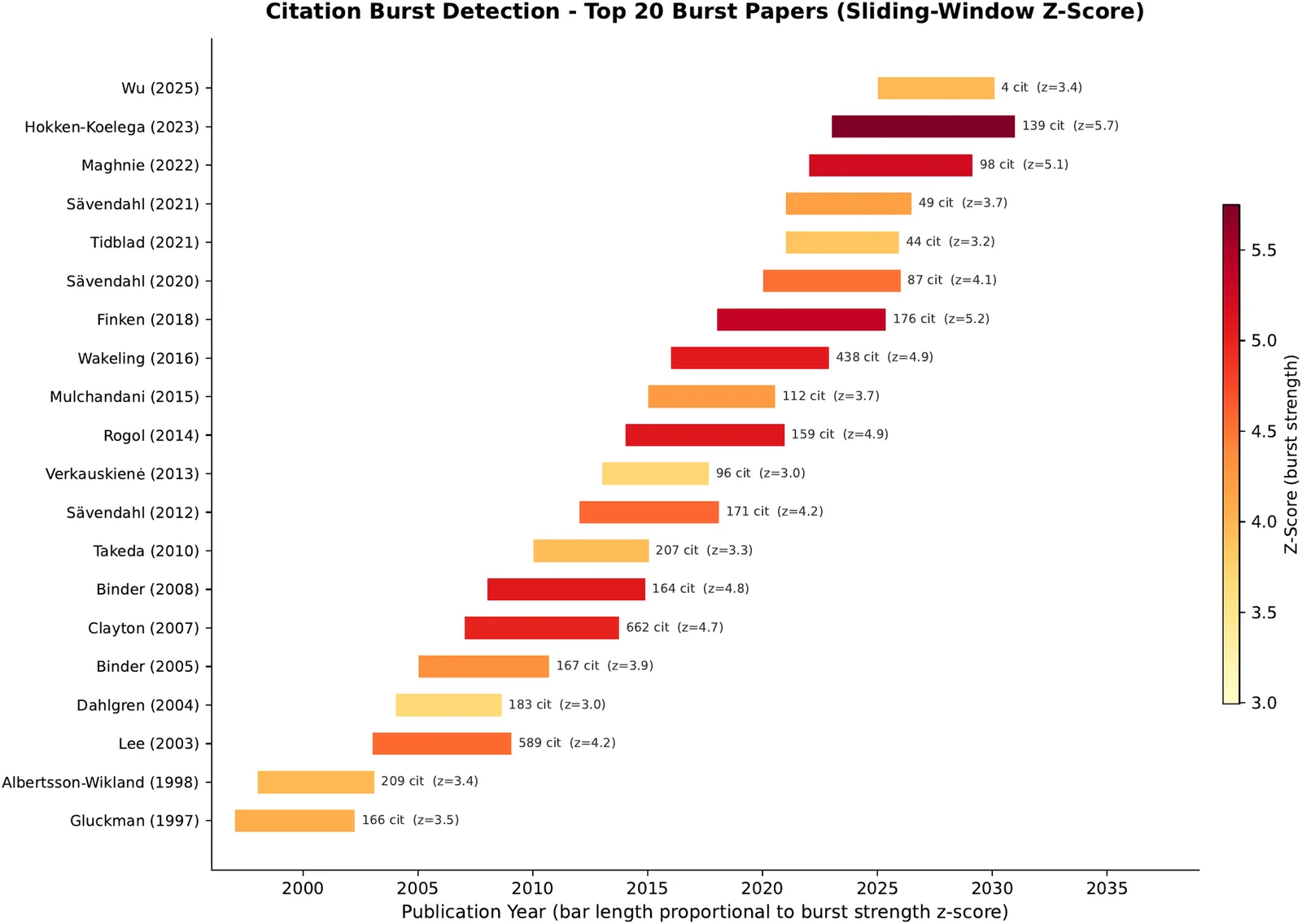
Citation burst detection using sliding-window Z-score method. Papers with citation-to-year-average ratio exceeding mean + 2 × SD are shown. Bar length is proportional to burst strength (Z-score).

Citation burst analysis (Figure 8) identified papers that received disproportionately high citations relative to their publication year. The strongest bursts were associated with major consensus statements: the SGA management guideline (2), the Silver–Russell syndrome consensus (29), and the molecular genetics review (10).

### Open access trends

**Figure 9:**
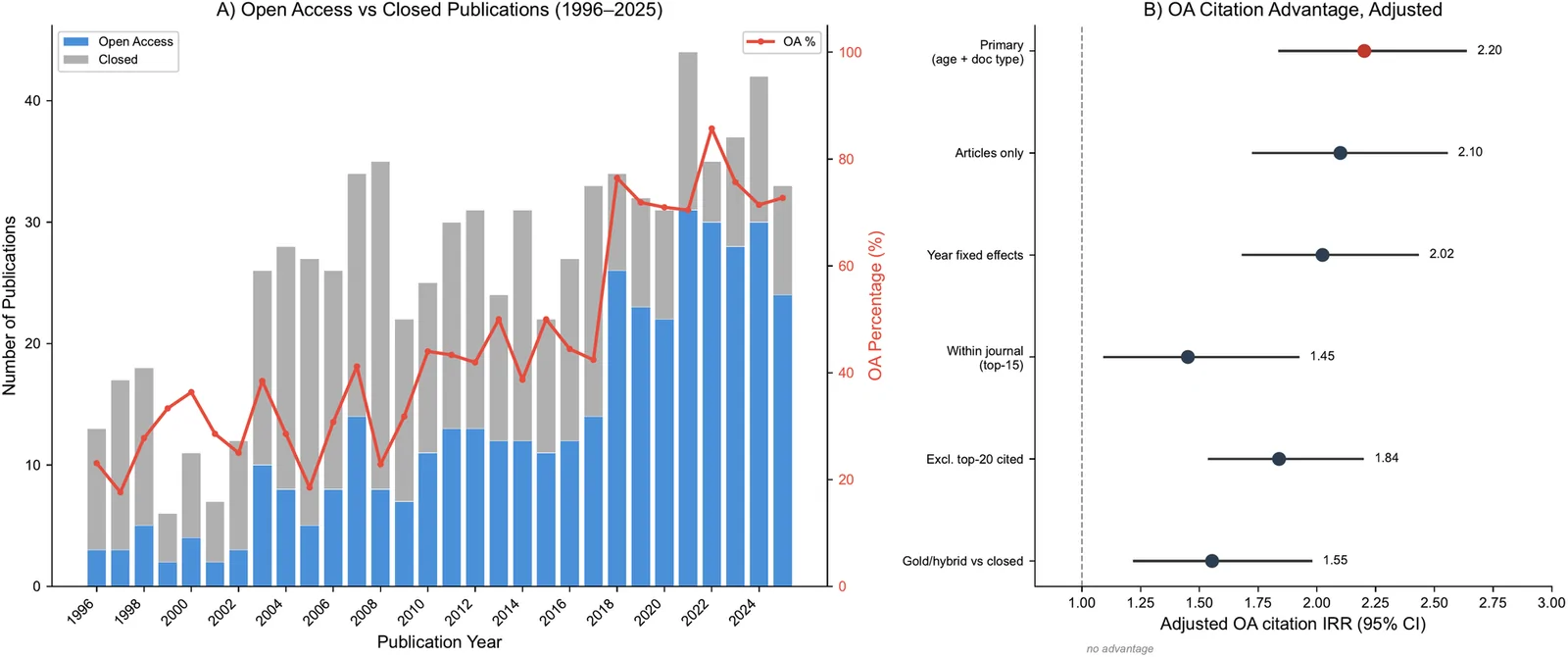
Open access in rhGH–SGA research. **(A)** OA versus closed-access publications by year, with the OA percentage trend line. **(B)** Adjusted OA citation incidence-rate ratios across model specifications (forest plot; all specifications lie above the null of 1).

Figure 9 presents the evolution of OA publishing in the rhGH–SGA literature. Of the 793 publications, 394 were available through some form of open access and 399 were classified as closed. The OA subtypes were gold (191), green (46), bronze (109), and hybrid (48). The OA proportion rose from approximately 17%–27% in the late 1990s to a peak of 85.7% in 2022, reflecting broader trends in biomedical publishing.

Because citation counts depend strongly on how long a paper has been in circulation and on its document type, the OA–citation comparison was adjusted for both. Open-access papers in this corpus were, on average, about six years more recent than closed-access papers (mean publication year 2016 vs. 2010) and less often reviews (13% vs. 22%), and reviews accrued roughly twice the citations of original articles (mean 41.8 vs. 22.1); both imbalances bias an unadjusted comparison against OA. The unadjusted Mann–Whitney U test was accordingly null (U = 79,171.5, *p* = 0.86; Cohen's d = 0.14). After adjustment in a negative-binomial model, open-access articles received 2.20-fold more citations than closed-access articles (95% CI 1.84–2.63, *p* < 0.001). The association was robust to excluding reviews (2.10), to publication-year fixed effects (2.02), to excluding the 20 most-cited papers (1.84), and to restricting open access to gold and hybrid routes (1.55); it attenuated but remained significant within journals (1.45, *p* = 0.009) and held in all four publication-era strata, and an age-normalised comparison (citations per year) agreed (*p* < 0.001). This is an association rather than a demonstrated causal effect—author self-selection, funding, and venue are not fully controlled—but its direction is opposite to the unadjusted null.

### Keyword trend analysis

**Figure 10:**
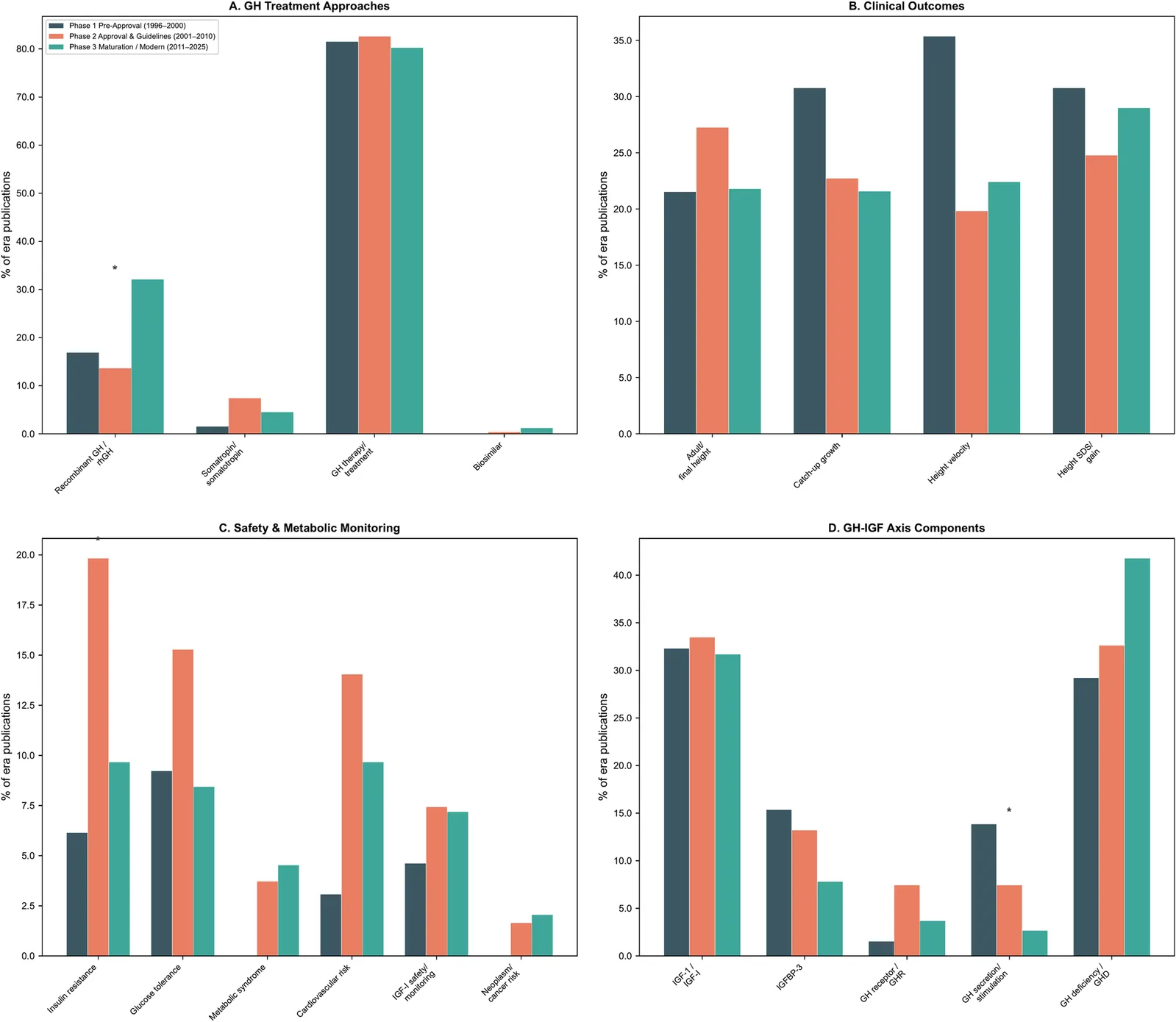
Keyword trend analysis: proportions of articles mentioning selected terms across the three regulatory phases (Phase 1, Pre-Approval, 1996–2000; Phase 2, Approval & Guidelines, 2001–2010; Phase 3, Maturation & Modern, 2011–2025). (**A**) GH treatment approaches; (**B**) clinical outcomes; (**C**) safety and metabolic monitoring; (**D**) GH–IGF axis components. Bars show the percentage of publications within each phase. Asterisks mark trends that remained significant after Benjamini–Hochberg correction.

Chi-square analysis of keyword frequencies across the three regulatory phases identified, of 37 terms tested, 8 with temporal trends that remained significant after Benjamini–Hochberg correction (Figure 10). The nine additional terms that reached nominal significance but did not survive correction are noted below as suggestive only.

Terminology shifts. The strongest effect was the decline of “IUGR” (intrauterine growth retardation) from 58.5% of articles in Phase 1 to 29.3% in Phase 2 and 15.3% in Phase 3 (*χ*^2^ = 66.9, q < 0.001), consistent with the international consensus shift from “IUGR” toward “SGA” and “fetal growth restriction.” Conversely, explicit use of “rhGH/recombinant GH” rose to 32.2% in Phase 3 (*χ*^2^ = 31.8, q < 0.001), reflecting the increasing specificity of therapeutic nomenclature.

Study methodology evolution. Observational and registry-based studies rose sharply across phases (*χ*^2^ = 37.6, q < 0.001), reflecting the maturation from efficacy trials to real-world evidence generation. Randomized controlled trials peaked during Phase 2 before declining in Phase 3 (*χ*^2^ = 14.1, q < 0.01), and dose-response studies followed the same pattern (*χ*^2^ = 15.7, q < 0.01), consistent with a transition from establishing efficacy to long-term surveillance.

GH-axis pharmacology. Terms for GH secretion/stimulation testing (*χ*^2^ = 19.1, q < 0.001), high-dose regimens (*χ*^2^ = 19.1, q < 0.001), and insulin resistance (*χ*^2^ = 17.9, q < 0.001) also showed significant cross-phase shifts, the last peaking in Phase 2 alongside early metabolic safety surveillance.

Suggestive but not surviving correction. Several clinically interesting terms reached nominal significance but did not survive multiplicity correction and are therefore reported as descriptive only: Silver–Russell syndrome (*χ*^2^ = 8.7, *p* = 0.013), GH deficiency (*χ*^2^ = 8.0, *p* = 0.018), IGFBP-3 monitoring (*χ*^2^ = 7.4, *p* = 0.025), and GH receptor/GHR mentions (*χ*^2^ = 6.6, *p* = 0.037).

### Topic modeling with BERTopic

BERTopic analysis of 771 abstracts identified 7 coherent research topics, with 605 documents assigned to topics and 166 (21.5%) classified as outliers not strongly assigned to any single topic. The topics, their sizes, and interpretive labels are presented in Table 4. This configuration recovered the same seven-topic solution across all ten random seeds tested, indicating a seed-robust solution.

**Table 4:** BERTopic research themes identified in rhGH–SGA literature.

Topic	Interpretive Label	*n*
1	GH treatment efficacy & adult height outcomes	391
2	GH–IGF-I axis & growth response	104
3	Silver–Russell syndrome	43
4	Genetic short stature (ACAN, CNVs, variants)	24
5	Treatment adherence & GH delivery devices	15
6	Experimental/animal models of IUGR	14
7	Neurocognitive & psychosocial outcomes in SGA	14

**Figure 11:**
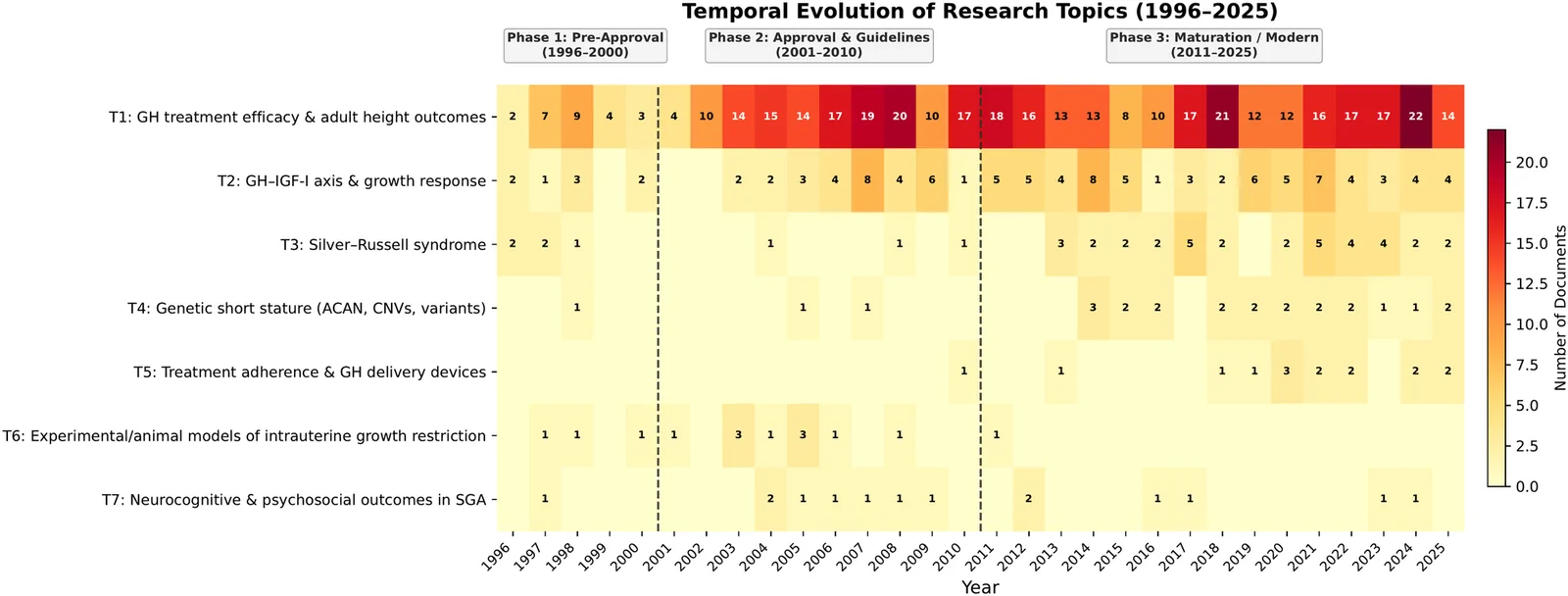
Temporal heatmap of BERTopic research themes (1996–2025). Cell intensity reflects the number of documents assigned to each topic per year.

Temporal topic evolution (Figure 11) showed shifts in research focus over the study period:

- Phase 1/Pre-Approval Era (1996–2000): the efficacy and adult-height theme (Topic 1) dominated, consistent with the field's early establishment of efficacy in the seminal multi-centre trials; the smaller molecular, genetic, and patient-centered themes were largely absent.
- Phase 2/Approval & Guidelines Era (2001–2010): Topic 1 remained predominant while the GH–IGF-I axis theme (Topic 2) and early genetic and metabolic work grew, reflecting post-approval interest in treatment response and safety.
- Phase 3/Maturation & Modern Era (2011–2025): although Topic 1 remained the leading theme, its relative share contracted as the corpus diversified toward molecular and genetic diagnostics (GH–IGF-I axis, Silver–Russell syndrome, genetic short stature including ACAN and copy-number variants) and patient-centered topics (treatment adherence and delivery devices, neurocognitive and psychosocial outcomes). Together these shifts reflect the field's transition toward precision medicine and patient-centered outcomes.

### Co-citation and bibliographic coupling

**Figure 12:**
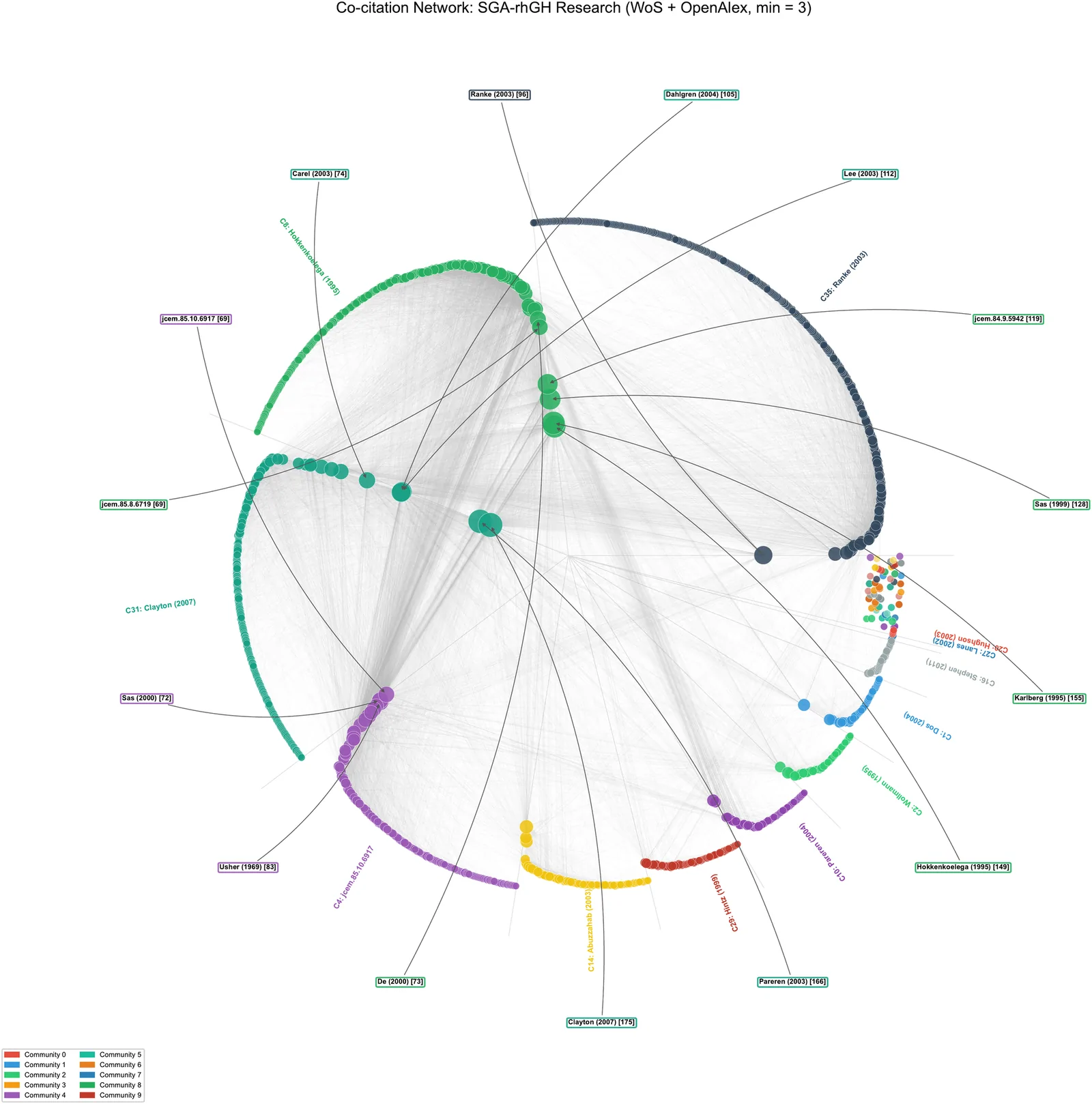
Co-citation network showing intellectual structure of rhGH–SGA research. Nodes represent frequently co-cited references; edges connect references that are cited together. Colors indicate Louvain communities.

Co-citation analysis combined reference data from Web of Science cited references and OpenAlex referenced_works, yielding 638 documents with 28,886 reference entries and 12,501 unique references. The co-citation network comprised 1,518 nodes and 16,870 edges (minimum co-citation threshold = 3), with 38 Louvain communities (Figure 12).

The three largest co-citation communities corresponded to early GH efficacy and dose-response studies [anchored by Ranke et al. 2003 (31–33)]; the Dutch SGA cohort and adult-height trials [anchored by van Pareren et al. 2003 (6), Hokken-Koelega et al. 1995 (34), Sas et al. 1999 (5)]; and consensus statements and SGA management [anchored by Clayton et al. 2007 (1), Lee et al. 2003 (3), Dahlgren 2005 (35), Saenger et al. 2007 (30)].

**Figure 13:**
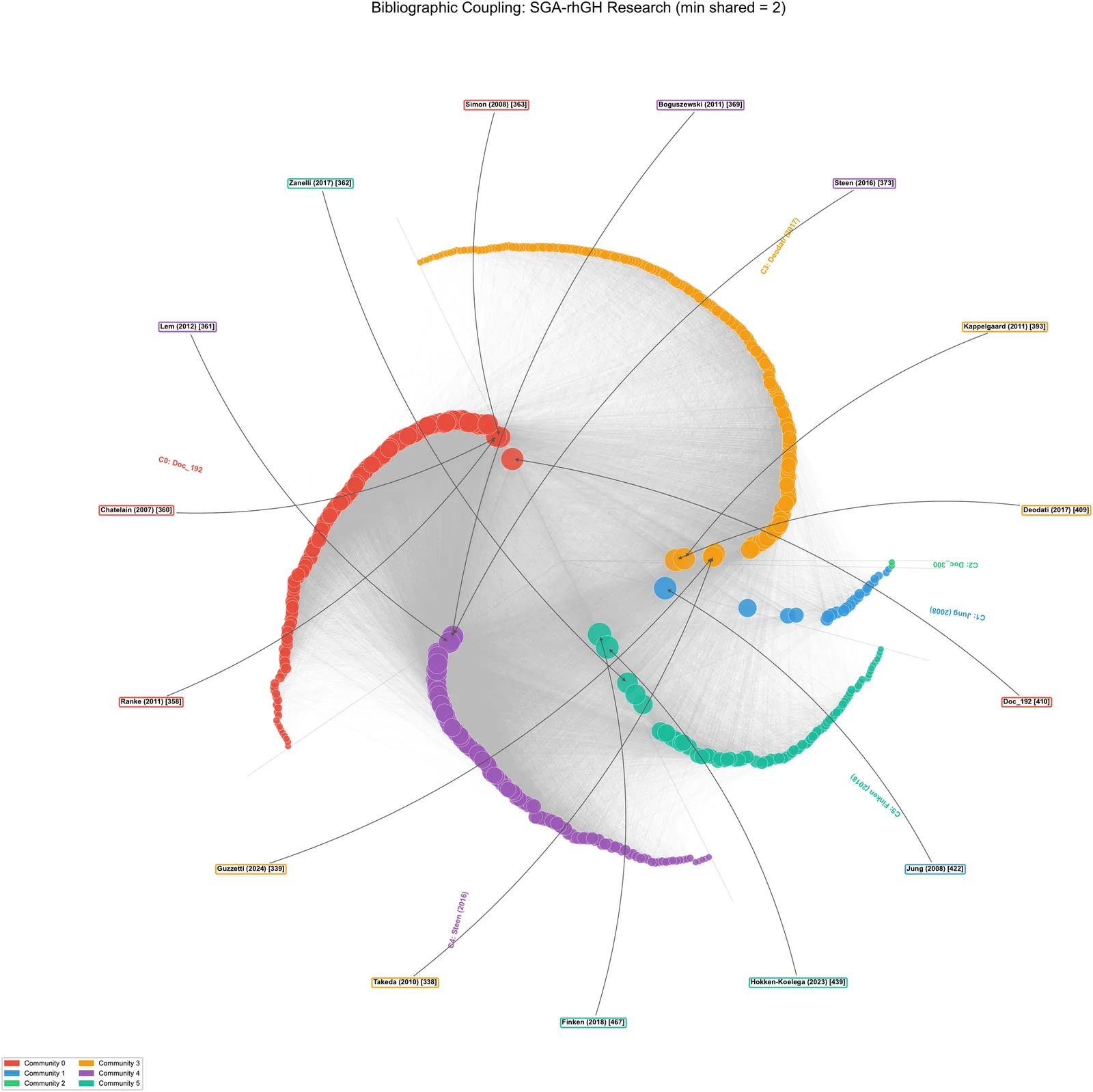
Bibliographic coupling network linking corpus documents that share cited references. Node size reflects document citation count; colors indicate Louvain communities.

Bibliographic coupling linked 591 corpus documents through 40,495 shared-reference edges (minimum shared references = 2) (Figure 13), confirming the clinical focus of the field, with bridging documents connecting the pharmacogenomic and clinical-efficacy communities.

## Discussion

### Landscape overview

The stabilized annual output and three-phase growth trajectory suggest a field that has largely addressed its foundational efficacy question—whether rhGH increases adult height in SGA children—and is increasingly engaging with precision-oriented questions. The temporal topic evolution (Figure 11) shows recent publications weighted toward molecular, genetic, and patient-centered themes, while the share of broad efficacy studies has plateaued. The emergence of the GH–IGF-I axis, Silver–Russell syndrome, and genetic short stature (ACAN and copy-number variants) as distinct BERTopic themes, alongside patient-centered clusters for treatment adherence and neurocognitive outcomes, is consistent with a shift toward identifying which patients benefit most and reframing success around patient-centered goals.

The concentration of publications in 4 core journals (Bradford Zone 1) suggests that clinicians can maintain field awareness by monitoring these venues, led by *Hormone Research in Paediatrics* and the *Journal of Clinical Endocrinology & Metabolism*.

### Authorship and collaboration patterns

Author productivity was highly concentrated, consistent with Lotka's Law. Hokken-Koelega ACS's dominance (81 publications) reflects her sustained involvement in the PROGRAM study (PROgramming factors for GRowth And Metabolism) (36), Dutch SGA cohorts, and key consensus documents. The co-authorship network shows a field structured around densely connected European research groups, with the Netherlands, Germany, and Scandinavia forming the core collaborative triangle.

The MCP ratio of 52.2% reflects the necessity of multi-center trials for this relatively uncommon indication. Japan's lower international connectivity (MCP ratio 22.6%) suggests a more domestically focused research program, likely influenced by Japan's distinct regulatory pathway and healthcare structure.

### Geographic patterns

The strong European dominance (7 of the top 10 countries) contrasts with many biomedical fields where the United States leads by a wider margin. Several hypotheses may explain this pattern, although our bibliometric data cannot test them directly. First, the EMA approved rhGH for SGA earlier than some other regulatory agencies (3), which may have stimulated European clinical research. Second, prolific European research groups—for example, Hokken-Koelega's Dutch SGA cohorts (6)—may have anchored collaborative networks around regional expertise. Third, European clinical trial registries, such as the KIGS database administered from Sweden (37), provided infrastructure for multi-center studies.

These patterns also distinguish the rhGH–SGA field from adjacent literatures. Recent bibliometric analyses of idiopathic short stature report rapidly rising output from China and East Asia (12, 13), and the broader short-stature and intrauterine-growth-restriction literatures show similarly diversifying geography (14, 15); by contrast, the rhGH–SGA field has remained concentrated in Western Europe and North America, likely reflecting its distinct regulatory history (early EMA approval) and its reliance on a small number of long-running European cohort and registry programmes.

The low representation of low- and middle-income countries remains a gap in the global evidence base. These geographic patterns should be interpreted cautiously, as affiliation data were available for 88.3% of the corpus.

### Keyword trends and thematic evolution

The keyword trend analysis provides a quantitative complement to BERTopic's semantic clustering by tracking measurable shifts in the field's vocabulary across the three regulatory phases. The dramatic decline of “IUGR” (*χ*^2^ = 66.9) and the simultaneous rise of “rhGH” terminology (*χ*^2^ = 31.8) document the field's evolving nomenclature, reflecting consensus recommendations to use “SGA” rather than “IUGR” and to specify the therapeutic agent precisely. All trends discussed here survived false-discovery-rate correction across the 37 tests performed.

The methodological shift from RCTs to observational/registry studies traces a classic maturation arc: early randomized trials established efficacy (5, 6, 9), leading to regulatory approval, which in turn generated the clinical uptake necessary for large observational cohorts and post-marketing surveillance. This transition is consistent with the KIGS (37), NordiNet (38), and GeNeSIS (8) registry programs and the SAGhE long-term mortality cohort (7) that now dominate long-term outcome data.

The 7 BERTopic themes trace a trajectory from basic efficacy questions toward molecular, genetic, and patient-centered investigations. The early literature addressed fundamental questions—does GH work in SGA, at what dose, and for how long (Topic 1). Over time the focus broadened to the GH–IGF-I axis and treatment response (Topic 2) and to genetic and patient-centered themes.

Several emerging themes deserve particular attention:

1. Genetic diagnostics (Silver–Russell syndrome, Topic 3; genetic short stature, Topic 4): as high-throughput sequencing and methylation testing have matured, imprinting disorders such as Silver–Russell syndrome and monogenic causes of short stature (e.g., ACAN variants, copy-number variants, IGF1R defects) have become identifiable BERTopic themes. This reflects the field's move toward molecular diagnosis of the heterogeneous SGA population.
2. The GH–IGF-I axis (Topic 2): IGF-I, IGFBP-3, IGF1R and GH-receptor signalling form a coherent molecular theme spanning treatment-response prediction and pharmacogenomics, a step toward individualized GH dosing in SGA.
3. Patient-centered outcomes (treatment adherence and delivery devices, Topic 5; neurocognitive and psychosocial outcomes, Topic 7): distinct themes centered on adherence-tracking injection devices and on cognitive and quality-of-life outcomes reflect growing engagement with patient-reported and functional endpoints alongside anthropometric ones.
4. Experimental models (Topic 6): a small but distinct cluster of animal and mechanistic studies of intrauterine growth restriction persists alongside the clinical literature, underpinning the biological rationale for treatment.

### Citation dynamics and open access

The Gini coefficient of 0.69 demonstrates substantial citation inequality, consistent with the Matthew effect (39) in clinical fields where guideline documents serve as obligatory passage points for subsequent research. To test whether this concentration is an artifact of self-citation, we examined the citation links internal to the corpus: 721 of 3,476 such links (20.7%) were author self-citations, a proportion typical of specialized clinical fields. Critically, the most-cited papers were not self-citation artifacts—the Clayton consensus, for example, received 150 internal citations of which only 12 (8%) were self-citations—so the concentration reflects the field's reliance on consensus anchors rather than self-referential inflation. This internal measure is bounded to within-corpus links; citations arriving from outside the corpus were not assessed.

Contrary to the unadjusted comparison, open-access articles in this corpus held a citation advantage once citation age and document type were accounted for (adjusted incidence-rate ratio 2.20; within-journal 1.45). The unadjusted null arose because open-access papers were newer and less often reviews—both properties that depress raw citation counts—so the two biases masked a real association. An open-access citation advantage of this magnitude is consistent with reports in several biomedical fields, although its causal interpretation remains debated. Greater accessibility, author self-selection of stronger work for open-access venues, and funding that supports both open-access fees and higher-impact research are all plausible contributors, and they cannot be separated with bibliometric data. The steady rise in open-access publishing (85.7% by 2022) nonetheless supports broader global accessibility.

### Methodological contribution

Our integration of BERTopic with classical bibliometric methods and keyword trend analysis illustrates how these approaches complement one another at three analytical levels. Keyword co-occurrence identifies broad thematic clusters from term co-occurrence; keyword trend analysis quantifies temporal shifts in specific terminology across the three regulatory phases; and BERTopic resolves finer-grained latent topics from full abstract semantics—for example distinguishing the Silver–Russell syndrome literature from broader genetic short-stature and GH–IGF-I-axis work that share vocabulary but address distinct questions. Combining frequency-based, temporal, and semantic approaches yields a more complete picture than any one method alone.

### Future directions

Several research directions emerge. First, AI and machine learning—of which transformer-based topic modelling is one example—are well placed to support individualised growth-response prediction, computer-vision–assisted bone-age estimation, and EHR-integrated dose-titration tools as registries mature into machine-readable cohorts.

Second, multi-omics genomics (whole-genome/exome sequencing, methylation arrays, transcriptomics) will refine SGA diagnostics, differentiating monogenic and imprinting disorders (Silver–Russell syndrome, IGF1R haploinsufficiency, PAPP-A2 deficiency) from polygenic short stature. Combined with broader pharmacogenomic risk scores beyond d3-GHR, this will enable personalised dosing strategies that integrate genotype, IGF-I/IGFBP-3 monitoring, predictive growth-response models, and patient preferences (daily vs. long-acting), evaluated in prospective comparative-effectiveness and pharmacoeconomic studies.

Third, real-world evidence and patient-reported outcomes [QoLISSY (40)] should be standardised alongside long-term cardiometabolic surveillance to reframe success around patient-centred goals. Finally, the European/North American concentration of the field highlights an equity gap: building research capacity, registry infrastructure, and treatment access in low- and middle-income countries—where SGA prevalence is higher—is essential, and future bibliometric updates should monitor whether this gap narrows.

### Limitations

This study has the following limitations:

1. Database coverage: while four databases were used (Lens.org, OpenAlex, Web of Science, and Scopus), each has its own indexing biases. Of the 793 final records, 425 (53.6%) were indexed in all four. We did not search PubMed or Embase directly; however, 85% of the corpus is PubMed-indexed, and the four sources were chosen because—unlike PubMed and Embase—they provide the citation counts, cited-reference lists, and structured affiliations required for citation, co-citation, coupling, and productivity analyses. Reference-based analyses covered 638 of 793 documents (80.5%).
2. Geographic data completeness: Country data were available for 88.3% of the corpus (from the OpenAlex subset, supplemented by affiliation parsing), potentially introducing selection bias in geographic analyses. Missing affiliations may be non-random: older publications and papers from lower-resource settings are less likely to have structured metadata in OpenAlex, which could lead to underestimation of contributions from these groups.
3. Language bias: Although no explicit language filter was applied, all four databases are biased toward English-language publications, potentially underrepresenting research from non-English-speaking regions (particularly East Asia and Latin America).
4. Citation and database snapshot: citation counts and open-access status represent a single time point (February 2026). OpenAlex in particular is continuously updated, so re-running these analyses at a later date would yield somewhat different counts; the reported figures should be read as a snapshot rather than a fixed value.
5. Author disambiguation: Manual name-variant merging was performed for the most prolific authors (Hokken-Koelega: 11 variants; de Zegher, Albertsson-Wikland, and Cianfarani: 2–3 variants each), but systematic automated disambiguation (e.g., ORCID-based resolution or machine-learning entity matching) was not applied across all authors. Consequently, some name fragmentation may persist for less prolific authors, particularly those with common Asian surnames or inconsistently hyphenated European names, potentially underestimating their true publication counts.
6. Keyword source: Keyword co-occurrence networks were based on OpenAlex concept tags (algorithmically assigned hierarchical subject labels) rather than author-supplied keywords. While concept tags offer higher coverage (92.4% for concept tags in this four-database corpus vs. typically 60%–75% for author keywords) and standardized vocabulary, they may miss emerging terms not yet in the classification taxonomy and may introduce noise from overly broad parent categories. The 37-term stopword list mitigated the latter, but some residual influence of the hierarchical tag structure may persist. Keyword trend analysis used title and abstract text directly, avoiding this limitation but potentially missing information contained only in keywords.
7. Screening approach: Record screening combined an automated relevance filter with a consensus expert audit rather than two independent, blinded reviewers; consequently, a formal inter-rater agreement statistic (e.g., Cohen's *κ*) could not be computed. Although all inclusion and exclusion decisions were verified by the study team with clinician sign-off, this consensus-based approach is more susceptible to reviewer subjectivity than a dual independent rating design.

## Conclusion

This bibliometric analysis of 793 publications (1996–2025) from four databases characterizes the rhGH–SGA research landscape. Key findings include: (1) a three-phase growth pattern temporally aligned with regulatory milestones; (2) strong European leadership with contributions from 67 countries (52.2% MCP); (3) substantial citation inequality (Gini = 0.69) that is not an artifact of self-citation; (4) an open-access citation advantage that emerges only after adjusting for citation age and document type (adjusted incidence-rate ratio 2.20), reversing the unadjusted null; (5) 8 keyword-trend shifts significant after multiplicity correction, documenting the transition from “IUGR” to “SGA” terminology and from randomized trials to observational/registry designs; and (6) a thematic diversification from efficacy studies toward molecular and genetic diagnostics and patient-centered outcomes, identified by BERTopic.

Future studies should extend BERTopic analyses to other specialized endocrinology fields and track the impact of long-acting and biosimilar GH formulations (16, 17, 41) on the field's thematic trajectory.

## Data Availability

All data and analysis code underlying this bibliometric study — including the merged 793-publication dataset, the co-authorship and keyword co-occurrence network files, the BERTopic model outputs, and the Python and R analysis scripts — are publicly available on GitHub at https://github.com/borean/sga-rhgh-biblio. Abstract texts are not redistributed owing to database licensing restrictions and can be retrieved by readers through the DOIs listed in the dataset.

## Supplementary material

Supplementary Figure S1 BERTopic Hyperparameter Sensitivity Analysis.

Supplementary Figure S2 Lotka’s Law fit of author productivity (*β* = 2.25, R² = 0.92, KS *p* = 0.300).

Supplementary Figure S3 Strategic thematic map (centrality vs. density) generated from keyword clusters.

Supplementary Table S1 Year-by-year publication counts and mean citations per article (1996–2025).

Supplementary Table S2 Top contributing countries with single-country (SCP) and multiple-country (MCP) publication counts.

Supplementary Text S1 Boolean query strings used across the four databases.

Supplementary Text S2 Statistical report.
