# Supplementary material for: Three decades of recombinant human growth hormone research in children born small for gestational age: a bibliometric analysis (1996–2025)
Source: Front Pediatr. 2026 Sep 9;14:1903046. doi: 10.3389/fped.2026.1903046 (PMC13597677; doi:10.3389/fped.2026.1903046)
Supplement: Supplementary file 1 [file Datasheet1.pdf]

Supplementary Material

Three Decades of Recombinant Human Growth Hormone Research in Children Born Small for Gestational Age: A Bibliometric Analysis (1996–2025)

Manuscript 1903046 · Frontiers in Pediatrics. All values were generated by the analysis scripts released with this study; the corpus comprises n = 793 publications spanning the 30 complete calendar years 1996–2025.

S1 Text. Boolean query strings used across the four databases

Searches were executed between 8 and 18 February 2026: Lens.org and OpenAlex on 8 February; Web of Science Core Collection and Scopus on 18 February. Queries combined the same two concept sets (SGA-related terms AND GH-treatment terms), with syntactic adaptations for each query language and restricted to journal articles and reviews published 1996–2026 (the search window only; the analysed corpus was subsequently truncated to the 30 complete years 1996–2025). The OpenAlex harvest additionally carried four terms absent from the other three queries; these are listed explicitly below.

Lens.org

```
((title:("small for gestational age" OR "SGA" OR "intrauterine growth retardation" OR
"IUGR" OR "fetal growth restriction")) OR (abstract:(same terms))) AND ((title:("growth
hormone" OR "somatotropin" OR "rhGH" OR "GH therapy")) OR (abstract:(same terms))) AND
publication_type:("journal article" OR "review") AND year_published:[1996 TO 2026]
```

OpenAlex

```
search="small for gestational age OR SGA OR born SGA OR intrauterine growth retardation OR
intrauterine growth restriction OR IUGR OR fetal growth restriction" AND search="growth
hormone OR somatotropin OR somatropin OR rhGH OR GH therapy OR GH treatment" AND
type:journal-article OR type:review AND from_publication_date:1996-01-01 AND
to_publication_date:2026-12-31
```

Web of Science Core Collection

```
TS=("small for gestational age" OR "SGA" OR "intrauterine growth retardation" OR "IUGR" OR
"fetal growth restriction") AND TS=("growth hormone" OR "somatotropin" OR "rhGH" OR "GH
therapy") AND DT=(Article OR Review) AND PY=1996-2026
```

Scopus

```
TITLE-ABS-KEY(("small for gestational age" OR "SGA" OR "intrauterine growth retardation" OR
"IUGR" OR "fetal growth restriction") AND ("growth hormone" OR "somatotropin" OR "rhGH" OR
"GH therapy")) AND (LIMIT-TO(DOCTYPE,"ar") OR LIMIT-TO(DOCTYPE,"re")) AND PUBYEAR > 1995
AND PUBYEAR < 2027
```

Terms present only in the OpenAlex query: “intrauterine growth restriction”, “born SGA”, “somatropin”, “GH treatment”.

Initial yields: Lens.org n = 1,184; OpenAlex n = 1,343; Web of Science n = 1,121; Scopus n = 1,589. After DOI-based merging, deduplication, therapeutic-relevance filtering, reprint and conference-abstract removal, two-stage expert relevance auditing, and exclusion of five partial-year 2026 records, the final corpus comprised 793 publications (Fig 1).
