# Supplementary material for: Three decades of recombinant human growth hormone research in children born small for gestational age: a bibliometric analysis (1996–2025)
Source: Front Pediatr. 2026 Sep 9;14:1903046. doi: 10.3389/fped.2026.1903046 (PMC13597677; doi:10.3389/fped.2026.1903046)
Supplement: Supplementary file 2 [file Datasheet2.pdf]

# S2 Text. Statistical Report

Normality Assessment, Test Selection Justification,
and Complete Statistical Results

Three Decades of Recombinant Human Growth Hormone Research in Children Born Small for Gestational Age: A Bibliometric Analysis (1996–2025) — $n = 793$

**Purpose of This Document**

This report documents the statistical methodology behind the manuscript:

1. **Normality testing** for citation distributions (to determine whether parametric tests are appropriate)
2. **Why specific tests were chosen** over alternatives
3. **Every statistical test result** referenced in the manuscript, with full detail
4. **Sensitivity analyses** to verify robustness of findings

## Contents


1 Normality Assessment of Citation Data 2

2 Statistical Test Selection: Why These Tests? 7

3 Complete Statistical Results 9

4 Sensitivity Analyses 13

5 Citation Burst Detection: Methodological Details 15

6 BERTopic Topic Modeling: Statistical Notes 16

7 Methodological Notes and Limitations 20

# 1 Normality Assessment of Citation Data

## 1.1 Full-Sample Tests

We tested the citation distribution of all 793 publications using three complementary normality tests. **All three tests overwhelmingly rejected the normality hypothesis** ($p < 10^{-20}$).

Table 1: Normality tests for the full citation distribution ($n = 793$)

| Test | Statistic | $p$-value | Normal? |
|---|---|---|---|
| Shapiro–Wilk | $W = 0.4557$ | $1.23 \times 10^{-43}$ | **No** |
| Kolmogorov–Smirnov | $D = 0.3111$ | $1.10 \times 10^{-68}$ | **No** |
| D'Agostino–Pearson | $K^2 = 1021.4$ | $1.63 \times 10^{-222}$ | **No** |

Table 2: Descriptive statistics for the citation distribution

| Measure | Value | Interpretation |
|---|---|---|
| $n$ | 793 | Total publications |
| Mean | 24.48 | Average citations per paper |
| Median | 9.0 | Typical paper; much lower than mean |
| Std. Dev. | 49.69 | Very large spread relative to mean |
| Min | 0 | Uncited papers |
| Max | 662 | Clayton et al. (2007) |
| Skewness | 6.654 | Extremely right-skewed ($\| > 2\| =$ severe) |
| Kurtosis | 65.000 | Extremely heavy-tailed ($\| > 7\| =$ severe) |
| % Uncited | 14.5% | 115 papers with zero citations |
| P25 | 2 | First quartile |
| P50 (median) | 9 | Second quartile |
| P75 | 27 | Third quartile |
| P90 | 57 | |
| P95 | 102 | |
| P99 | 185 | Top 1% of papers |

**Interpretation:** The citation distribution is *extremely* non-normal, with a skewness of 6.65 (normal = 0) and kurtosis of 65.0 (normal = 0). The mean (24.48) is roughly 2.7 times the median (9.0), a hallmark of right-skewed distributions. This pattern is universal in citation analysis and reflects the well-known "rich get richer" phenomenon (Matthew effect): landmark consensus statements and reviews accumulate citations at a rate far exceeding that of typical research papers.
**Bottom line:** The data are profoundly non-normal. Any parametric test assuming a bell-shaped distribution would be grossly inappropriate and would produce unreliable results. Non-parametric methods are required for all inferential analyses.

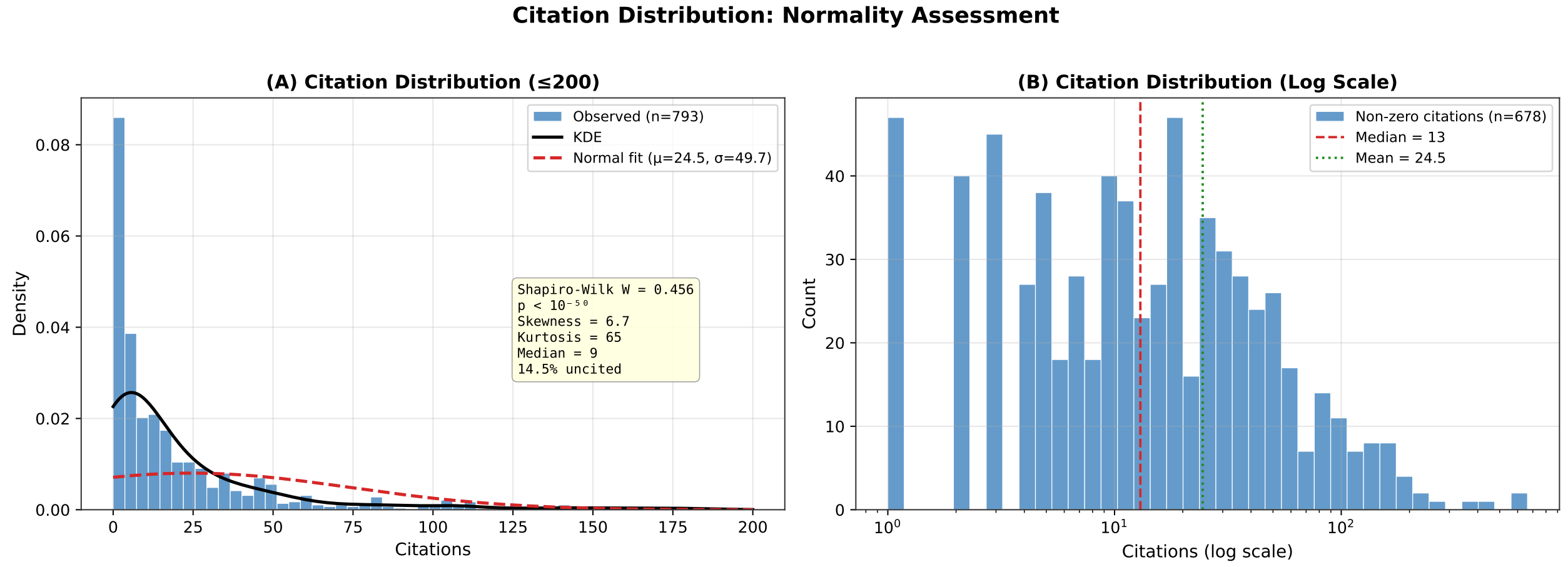


Figure 1: **Citation distribution diagnostics.** (A) Histogram of citations (≤200 shown) with kernel density estimate (KDE, black) and normal distribution fit (red dashed). The massive gap between the observed distribution and the normal curve visually demonstrates the extreme right-skew. (B) Log-scale view revealing the heavy-tailed, power-law-like distribution. The mean (green dotted) is roughly 3× the median (red dashed), a hallmark of non-normal data.

## 1.2 Subgroup Normality: OA vs. Closed-Access Papers

Since the primary inferential analysis compares citations between OA ($n = 394$) and closed-access ($n = 399$) papers (records with publication year 1996–2025), we tested normality within each subgroup.

Table 3: Subgroup normality assessment: OA vs. Closed-access citation distributions

| **Subgroup** | $n$ | $W$ | $p$ **(S-W)** | $D$ | $p$ **(K-S)** | **Skew** | **Normal?** |
|---|---|---|---|---|---|---|---|
| OA | 394 | 0.4711 | $1.70 \times 10^{-32}$ | 0.3142 | $4.52 \times 10^{-35}$ | 5.736 | **No** |
| Closed | 399 | 0.4562 | $5.17 \times 10^{-33}$ | 0.2994 | $3.29 \times 10^{-32}$ | 8.107 | **No** |

Table 4: Descriptive statistics by OA status

| **Group** | $n$ | **Mean** | **Median** | **SD** | **Skew** | **Kurtosis** |
|---|---|---|---|---|---|---|
| OA | 394 | 27.90 | 9.0 | 57.65 | 5.736 | 47.217 |
| Closed | 399 | 21.11 | 10.0 | 40.12 | 8.107 | 101.402 |

**Interpretation:** Both subgroups are profoundly non-normal. The closed-access group is even more skewed (8.11 vs. 5.74) and leptokurtic (101.4 vs. 47.2) than the OA group, largely driven by a small number of highly cited consensus statements. For a valid $t$-test, **both** groups must be normally distributed. Since both groups dramatically fail every normality test, the Mann–Whitney $U$ test is the only appropriate choice for comparing these groups.

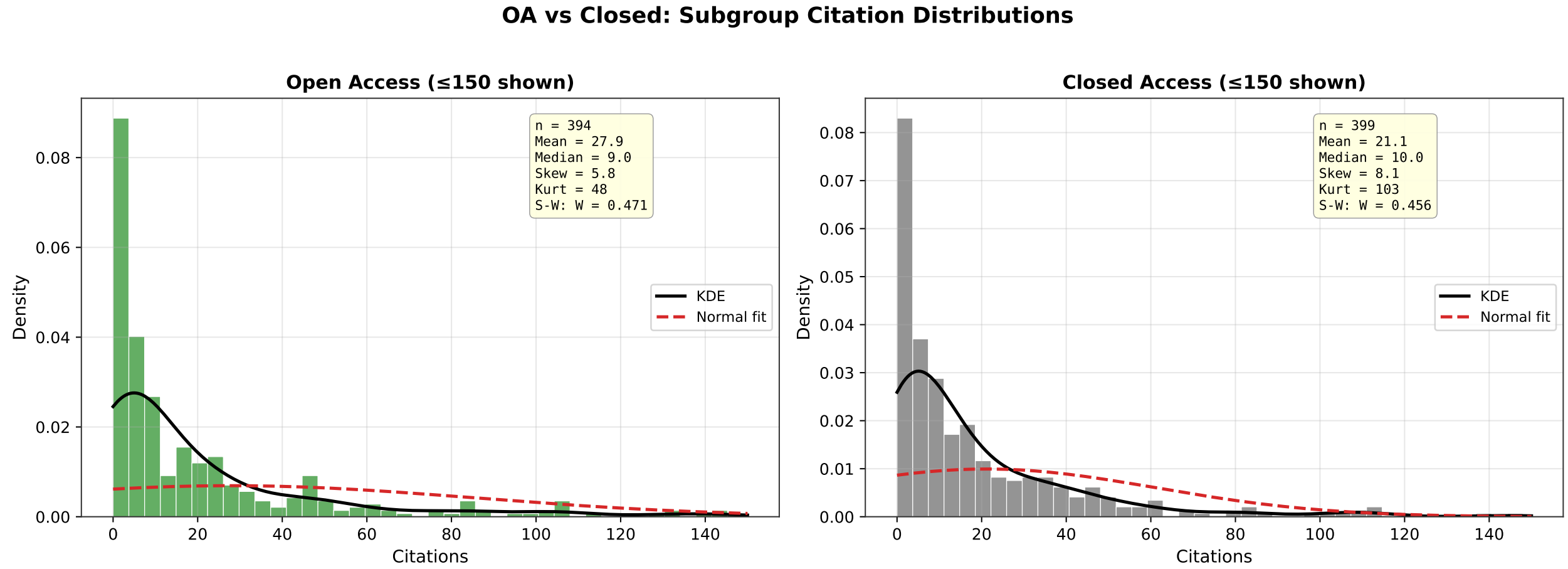


Figure 2: **OA vs. Closed subgroup citation distributions.** Both groups show extreme right-skew with the KDE (black) deviating massively from the normal fit (red dashed). The closed-access group (right) is even more skewed (8.1 vs. 5.7) due to a small number of highly cited consensus statements. Summary statistics (insets) confirm non-normality in both subgroups.

## 1.3 Log-Transformed Citation Data

We additionally assessed whether log-transformation ($\ln(\text{citations}+1)$) could normalize the data, as is sometimes attempted in citation analysis.

Table 5: Normality of log-transformed citations

| **Sample** | $n$ | $W$ | $p$ **(S-W)** | **Skew** | **Normal?** |
|---|---|---|---|---|---|
| Full corpus (log) | 793 | 0.9672 | $2.41 \times 10^{-12}$ | 0.036 | **No** |
| OA (log) | 394 | 0.9673 | $1.04 \times 10^{-7}$ | 0.132 | **No** |
| Closed (log) | 399 | 0.9621 | $1.24 \times 10^{-8}$ | −0.096 | **No** |

**Interpretation:** While log-transformation dramatically improved the skewness (from 6.65 to 0.04 for the full sample), the Shapiro–Wilk test still rejected normality at the $p < 10^{-7}$ level in every subgroup. This is partly because 14.5% of the data are zeros (uncited papers), creating a "zero-inflation" that no standard transformation can resolve. The residual non-normality, combined with the zero-inflated structure, means that even log-transformed parametric tests would not be fully valid. We nonetheless report a Welch's $t$-test on log-transformed data as a sensitivity analysis (Section 4).

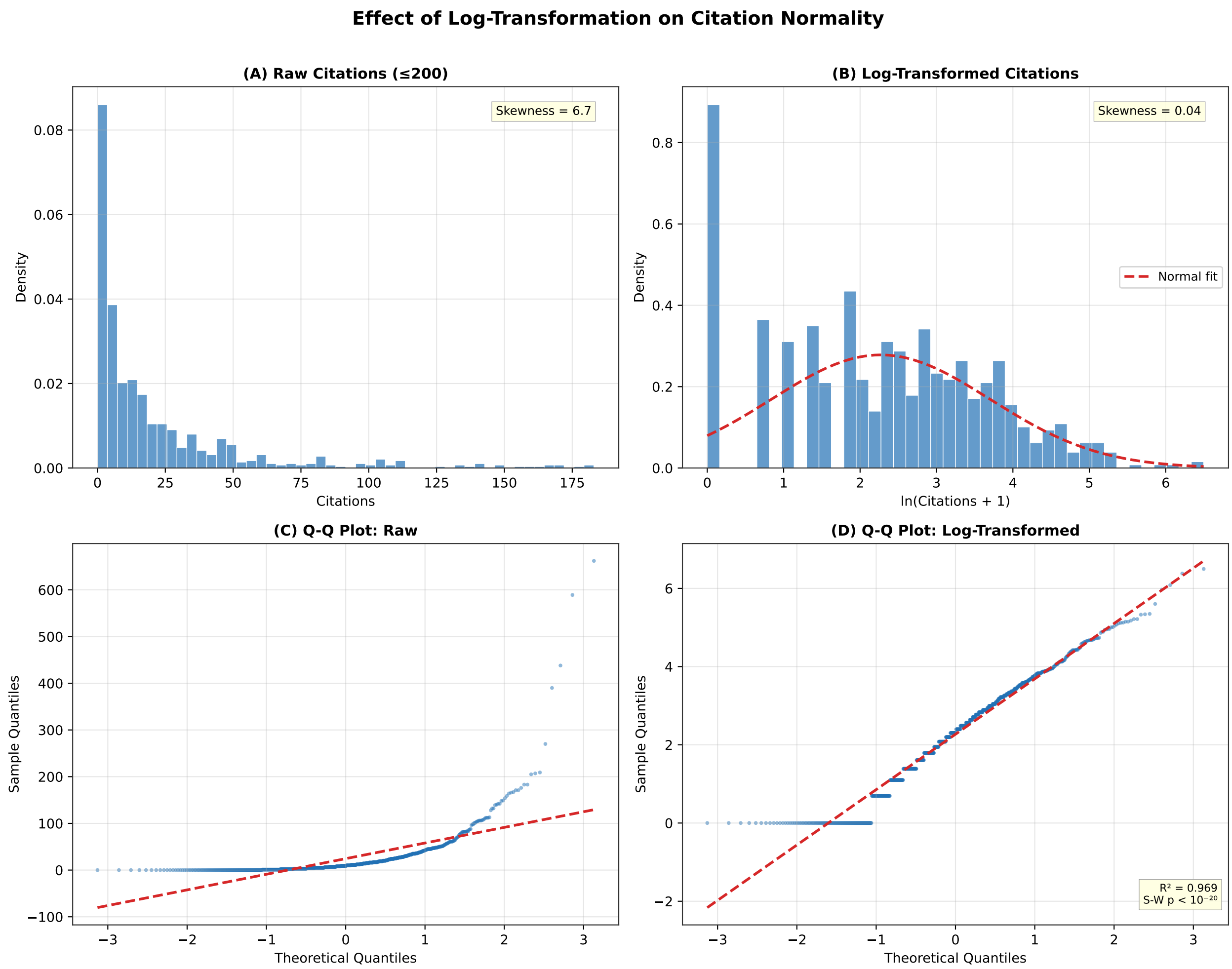


Figure 3: **Effect of log-transformation on citation normality.** Top row: raw citations (A) vs. log-transformed (B) histograms. Log-transformation dramatically reduces skewness (6.65 → 0.04) and visually improves the fit to a normal curve (red dashed), but the Shapiro–Wilk test still rejects normality ($p < 10^{-12}$). Bottom row: Q-Q plots for raw (C) and log-transformed (D) data. The raw Q-Q plot shows extreme deviation from the diagonal (the most-cited paper reaches 662 citations), while the log-transformed Q-Q plot is substantially improved but still shows systematic S-shaped deviation in the tails, confirming residual non-normality.

## 1.4 Q-Q Plot Diagnostics

Quantile–quantile (Q-Q) plots provide a visual complement to the formal normality tests. If data are normally distributed, points should fall along the diagonal reference line. Systematic deviations indicate non-normality.

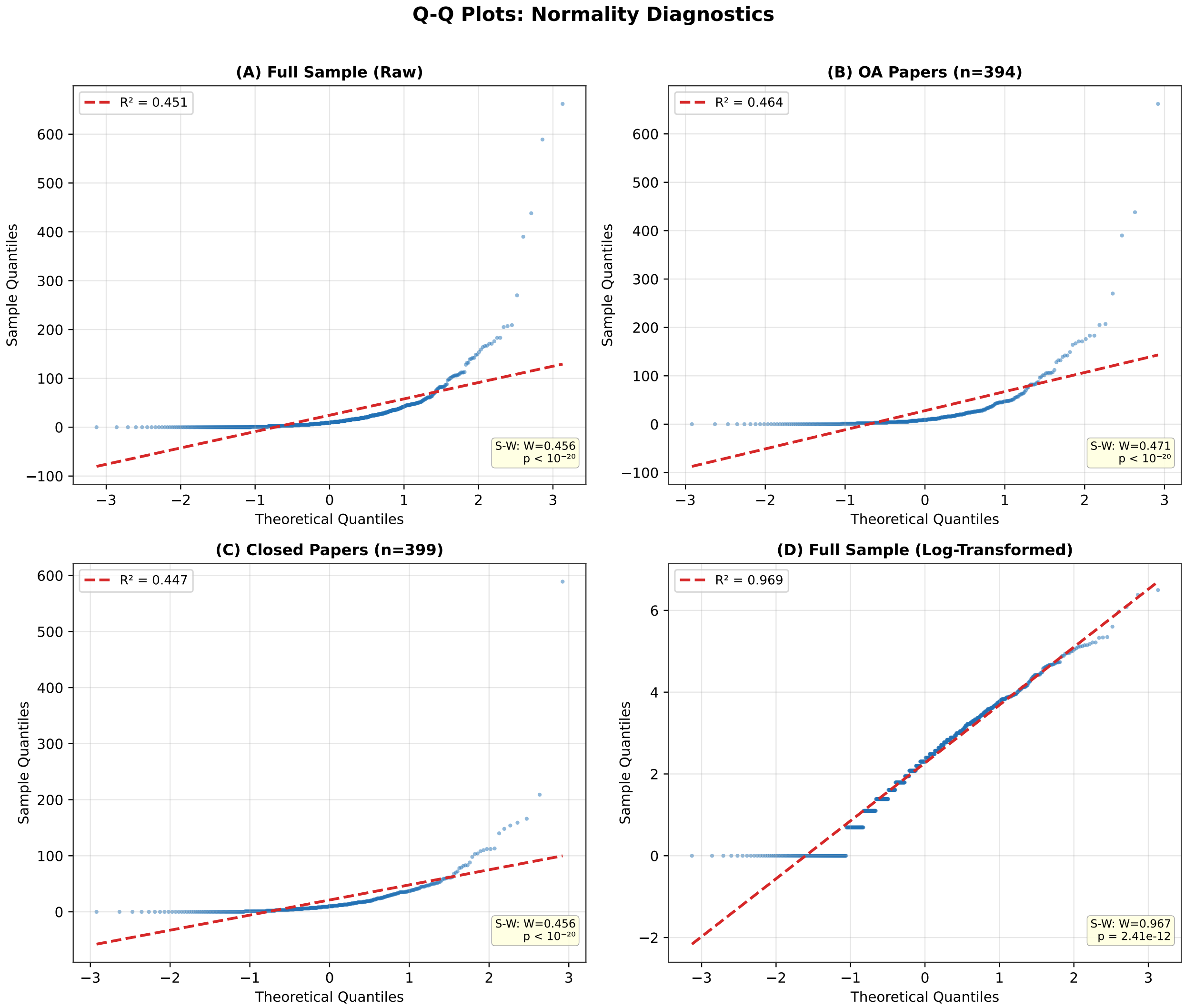


Figure 4: **Q-Q plots for normality diagnostics.** (A) Full sample: extreme upward curvature at the right tail demonstrates the heavy-tailed distribution. (B) OA papers: similar heavy-tail deviation. (C) Closed-access papers: comparable curvature driven by highly cited consensus papers. (D) Log-transformed full sample: substantially improved—points follow the diagonal more closely, but the S-shaped deviation in both tails confirms that even log-transformation does not fully normalize citation data. Shapiro–Wilk test results are shown in each panel.

## 2 Statistical Test Selection: Why These Tests?

Based on the normality assessment above and the characteristics of our data, we selected the following tests:

Table 6: Statistical tests used and justification

| Test used | Applied to | Why this test? |
|---|---|---|
| **Mann–Whitney $U$** | OA vs. Closed citation comparison | • Non-normal distributions confirmed (Table 1)<br>• Robust to extreme outliers (max = 662)<br>• Valid regardless of distribution shape<br>• Standard choice in citation analysis |
| **Gini coefficient** | Citation inequality measurement | • Standard inequality metric (0–1 scale)<br>• Does not assume any distribution<br>• Widely used in bibliometric studies |
| **Sliding-window Z-score** | Citation burst detection | • Year-normalized: accounts for field growth<br>• Z-score threshold ($\mu + 2\sigma$) is robust<br>• Custom implementation replacing CiteSpace |
| **Bradford's Law** | Journal productivity zones | • Classical bibliometric law<br>• Rank-frequency analysis (no distributional assumptions) |
| **Lotka's Law** ($\beta$ fit) | Author productivity distribution | • Inverse power law: $P(n) \propto n^{-\beta}$<br>• K-S goodness-of-fit test applied |
| **Louvain algorithm** | Network community detection | • Modularity-based community detection<br>• Scalable to large networks<br>• No distributional assumptions |

## 2.1 Tests We Deliberately Did NOT Use

Table 7: Parametric alternatives considered and rejected

| Test | Requirement | Why it fails for our data |
|---|---|---|
| Student's $t$-test | Normal distributions in both groups | Grossly violated: $W = 0.47$ (OA) and $W = 0.46$ (Closed), skewness 5.7 and 8.1 respectively (Tables 3–4) |
| Welch's $t$-test (raw data) | Approximate normality or large $n$ | Skewness $> 5$ even at $n > 500$ makes the CLT approximation unreliable; zero-inflation (15.0%) further distorts the mean |
| Pearson correlation | Bivariate normality | Violated for any citation-based variable; Spearman's $\rho$ would be appropriate but is not applied in this study |
| ANOVA / Kruskal–Wallis | ≥3 groups | Not applicable: OA comparison involves only 2 groups |

**Central Limit Theorem.** Large $n$ does not license a $t$-test here. CLT convergence is fast enough to rely on only for moderately non-normal distributions (skewness $< 2$); at the skewness ($> 6$) and kurtosis ($> 65$) observed in this corpus, the sampling distribution of the mean converges slowly, and simulation work in the citation-analysis literature places the requirement above $n = 1{,}000$. Rank-based inference avoids the issue.

# 3 Complete Statistical Results

## 3.1 Open Access and Citations: Confounder-Adjusted Analysis (Primary)

**Why the unadjusted comparison is not the primary analysis.** Citation counts depend on how long a paper has been in circulation and on its document type, and both are unbalanced by open-access status in this corpus. Open-access papers are on average 5.8 years more recent (mean publication year 2015.9 vs. 2010.1) and are less often reviews (13.5% vs. 21.6%), while reviews accrue roughly twice the citations of original articles (mean 41.8 vs. 22.1). These two biases act in opposite directions and very nearly cancel, which is what produces the null in the unadjusted test reported below.

We therefore estimate the association with a negative-binomial regression of citation count on open-access status, adjusting for citation age (ln years since publication) and document type. Document type was recovered by DOI from the four source databases (92.2% coverage).

Table 8: Negative-binomial incidence-rate ratios for open access, across specifications

| Specification | $n$ | IRR | 95% CI | $p$ |
|---|---|---|---|---|
| **Primary** (age + document type) | 731 | 2.20 | 1.84–2.63 | $< 0.001$ |
| Articles only (reviews excluded) | 592 | 2.10 | 1.73–2.55 | $< 0.001$ |
| Publication-year fixed effects | 731 | 2.02 | 1.69–2.43 | $< 0.001$ |
| Within-journal (journal fixed effects) | 404 | 1.45 | 1.09–1.92 | 0.009 |
| Excluding 20 most-cited papers | 711 | 1.84 | 1.54–2.19 | $< 0.001$ |
| Gold/hybrid OA only | 573 | 1.55 | 1.22–1.98 | $< 0.001$ |

Table 9: Age-normalised citations per year, by publication era

| Era | $n$ OA | $n$ closed | Median OA | Median closed | $p$ |
|---|---|---|---|---|---|
| 1996-2000 | 17 | 48 | 2.27 | 0.69 | 0.010 |
| 2001-2010 | 76 | 166 | 1.76 | 0.71 | $< 0.001$ |
| 2011-2017 | 87 | 111 | 1.38 | 0.77 | 0.002 |
| 2018-2025 | 214 | 74 | 0.94 | 0.71 | 0.024 |

Across the whole corpus the age-normalised comparison gives median 1.211 citations/year for open-access papers versus 0.71 for closed ($U = 95,004.0$, $p < 0.001$).

**Interpretation:** After adjustment, open-access articles received 2.20 times the citations of closed-access articles (95% CI 1.84–2.63, $p < 0.001$). The association survives excluding reviews, publication-year fixed effects, dropping the 20 most-cited papers, and restricting open access to the gold and hybrid routes. It attenuates substantially within journals (1.45, $p = 0.009$), which is what one expects if part of the effect operates through venue choice, and it holds in all four era strata.

**This is an association, not a causal effect.** Author self-selection of stronger work for open-access venues, funding that supports both article-processing charges and higher-impact research, and venue prestige cannot be separated with bibliometric data alone.

### 3.2 Mann–Whitney $U$ Test: Unadjusted Comparison (Secondary)

The unadjusted rank comparison makes no allowance for citation age or document type. It is reported for completeness and for comparability with the previously circulated version of this analysis; the adjusted model in Section 3.1 is the primary result.

Table 10: Mann–Whitney $U$ test: OA vs. Closed-access citation comparison

| Parameter | Value | Interpretation |
|---|---:|---|
| $n_{\text{OA}}$ | 394 | Papers with any OA status (1996–2025) |
| $n_{\text{Closed}}$ | 399 | Closed-access papers (1996–2025) |
| OA mean citations | 27.90 | Higher mean, but driven by outliers |
| OA median citations | 9.0 | Typical OA paper: 9 citations |
| Closed mean citations | 21.11 | |
| Closed median citations | 10.0 | Typical closed paper: 10 citations |
| $U$ statistic | 79,171.5 | |
| $p$-value | 0.860 | **Not significant** |
| *Effect sizes* | | |
| Rank-biserial $r$ | −0.007 | Negligible ($\|r\| < 0.1$ = negligible) |
| Cohen's $d$ | 0.137 | Negligible ($d < 0.2$ = negligible) |

**Interpretation:** Taken on its own, this test shows **no significant difference** in raw citations between OA and closed-access papers ($p = 0.860$), with negligible effect sizes (rank-biserial $r = -0.007$, Cohen's $d = 0.137$).
That null is an artifact of confounding rather than evidence of no association. The open-access papers in this corpus are markedly younger and less review-heavy than the closed-access ones; the first depresses their raw counts and the second inflates the comparison group's, and the two effects very nearly cancel. Once both are adjusted for (Section 3.1), a substantial open-access advantage emerges.

### 3.3 Gini Coefficient and Citation Inequality

Table 11: Gini coefficient sensitivity analysis

| Analysis | Gini | Interpretation |
|---|---:|---|
| Full corpus ($n = 793$) | 0.689 | High inequality |
| Excluding top 5 most cited | 0.6574 | Still high |
| Excluding top 10 most cited | 0.6472 | Still high |
| *Context* | | |
| Perfect equality (all papers equally cited) | 0.000 | Theoretical minimum |
| Perfect inequality (one paper gets all cites) | 1.000 | Theoretical maximum |
| Typical biomedical field | 0.6–0.8 | Literature range |

**Interpretation:** The Gini coefficient of 0.689 indicates that citation inequality is a *structural* feature of the rhGH–SGA literature, not an artifact of a few landmark papers. Even after excluding the top 10 most-cited papers, the Gini remains firmly in the "high inequality" range. This is the Matthew effect in action: consensus statements and reviews serve as "obligatory passage points" that accumulate citations far beyond typical research articles.

## 3.4 Bradford's Law of Journal Scattering

Table 12: Bradford zone distribution

| Zone | Journals | Articles | % of Total | Description |
|---|---|---|---|---|
| Zone 1 (Core) | 4 | 272 | 34.3% | Core journals |
| Zone 2 (Related) | 34 | 261 | 32.9% | Related journals |
| Zone 3 (Peripheral) | 202 | 260 | 32.8% | Peripheral journals |
| **Total** | 240 | 793 | 100% | |

**Interpretation:** The distribution follows Bradford's Law: 4 core journals produce roughly one-third of all output, while 202 peripheral journals produce the other third. The Bradford multiplier ($k$) between zones is approximately $k_1 = 34/4 = 8.5$. One-third of the rhGH–SGA literature is therefore captured by four journals, led by *Hormone Research in Paediatrics* ($n = 107$).

## 3.5 Lotka's Law of Author Productivity

Table 13: Lotka's Law parameters

| Parameter | Value | Interpretation |
|---|---|---|
| Total unique authors | 2,985 | |
| Single-publication authors | 79.8% | Close to Lotka's theoretical 60% |
| Lotka exponent ($\beta$) | 2.25 | $P(n) \propto n^{-2.25}$ |
| $R^2$ (goodness of fit) | 0.92 | Good fit |
| K-S $p$-value | 0.300 | Fit not rejected ($p > 0.05$) |
| *Reference* | | |
| Classical Lotka ($\beta = 2.0$) | — | Theoretical value |
| Observed $\beta > 2.0$ | — | Steeper than classical = more concentrated |

**Interpretation:** The author productivity distribution follows Lotka's inverse power law ($R^2 = 0.92$), with $\beta = 2.25$ slightly exceeding the classical exponent of 2.0. This means productivity is slightly more concentrated than the classical prediction: a few prolific researchers (e.g., Hokken-Koelega with 75 publications combining 11 name variants) dominate the field, while ∼80% of authors contributed only a single paper. The 79.8% single-author rate exceeds Lotka's theoretical prediction of ∼60%, which is common in specialized medical fields where many clinicians publish occasional case reports or collaborative studies.

# 4 Sensitivity Analyses

## 4.1 Welch's $t$-test on Log-Transformed Citations

Although normality was rejected even after log-transformation (Table 5), we report Welch's $t$-test on $\ln(\text{citations} + 1)$ as a sensitivity analysis to demonstrate that our conclusion does not depend on the choice of test.

Table 14: Sensitivity analysis: Welch's $t$-test on log-transformed citations

| Parameter | Value | Note |
|---|---|---|
| $t$ statistic | 0.5076 | |
| $p$-value | 0.612 | **Not significant** |

**Interpretation:** Even using a parametric test on log-transformed data (which favors detecting multiplicative differences), the result is not significant ($p = 0.612$). This confirms that the null finding from the Mann–Whitney $U$ test ($p = 0.860$) is robust and not an artifact of the chosen test.

## 4.2 Bootstrap Confidence Interval for Median Difference

As a distribution-free alternative to both the Mann–Whitney $U$ and $t$-tests, we computed a bootstrap 95% confidence interval for the difference in medians (OA − Closed), using 10,000 resamples.

Table 15: Bootstrap analysis of median citation difference

| Parameter | Value | Interpretation |
|---|---|---|
| Observed median difference | −1.0 | OA median lower |
| Bootstrap 95% CI | [−4.0, +2.0] | Includes zero |
| Zero in CI? | **Yes** | No significant difference |
| Number of bootstrap samples | 10,000 | |

**Interpretation:** The bootstrap 95% CI for the median difference spans −4.0 to +2.0, firmly including zero. This provides a third independent confirmation (alongside Mann–Whitney $U$ and the log-transformed $t$-test) that there is no meaningful citation advantage for OA papers in the rhGH–SGA literature.

### 4.3 Summary: What Each Approach Answers

Table 16: Unadjusted approaches agree with each other; adjustment changes the answer

| Method | Type | $p$-value | Conclusion |
|---|---|---|---|
| *Unadjusted (secondary)* | | | |
| Mann–Whitney $U$ | Non-parametric | 0.860 | Not significant |
| Welch's $t$-test (log data) | Parametric (approx.) | 0.612 | Not significant |
| Bootstrap median difference | Resampling | CI includes 0 | Not significant |
| *Adjusted (primary)* | | | |
| Negative binomial, age + type | Regression | $< 0.001$ | IRR 2.20 |
| Age-normalised citations/year | Non-parametric | $< 0.001$ | OA higher |

**Bottom line:** The three unadjusted approaches agree with one another, which is unsurprising—they are three ways of asking the same unadjusted question of the same data, so their agreement is not independent confirmation. What separates them from the adjusted analysis is not method but specification. Once citation age and document type enter the model, the direction reverses and open-access articles show 2.20 times the citations (95% CI 1.84–2.63). The adjusted estimate is the one reported in the manuscript; the unadjusted tests are retained here as context and as a record of why the earlier null arose.

# 5 Citation Burst Detection: Methodological Details

## 5.1 Sliding-Window Z-Score Method

Traditional citation burst analysis (e.g., Kleinberg's algorithm in CiteSpace) was not available due to software constraints. We implemented a custom sliding-window Z-score method:

1. For each publication year $y$, compute the mean ($\mu_y$) and standard deviation ($\sigma_y$) of citations for all papers published in year $y$.
2. For each paper $i$ published in year $y$, compute: $z_i = \frac{c_i - \mu_y}{\sigma_y}$
3. Papers with $z_i > 2$ are classified as "burst" publications.

**Year normalization.** Citation-to-year-average ratios are used rather than raw counts, so that burst detection reflects performance relative to contemporaries rather than time in circulation. Without it the method would favour older papers, whose absolute counts are higher because the field was smaller and the accumulation window longer.

**Validation:** The top burst papers identified by this method (Wakeling 2016, Hokken-Koelega 2023, Maghnie 2022, Clayton 2007) are all widely recognized landmark publications in the SGA field, supporting the method's face validity.

# 6 BERTopic Topic Modeling: Statistical Notes

## 6.1 Model Parameters and Rationale

Table 17: BERTopic hyperparameter selection

| Parameter | Value | Rationale |
|---|---|---|
| Embedding model | all-MiniLM-L6-v2 | Fast, accurate sentence embeddings (384-dim) |
| UMAP n_components | 5 | Balance between cluster coherence and information |
| UMAP n_neighbors | 15 | Default; captures local structure |
| UMAP min_dist | 0.0 | Tight clusters for HDBSCAN |
| UMAP metric | cosine | Standard for text embeddings |
| HDBSCAN min_cluster | 10 | Lowered from 15 for the 771-abstract corpus |
| HDBSCAN min_samples | 3 | Lowered from 5; yields a seed-stable solution |
| Vectorizer | English stopwords, min_df = 2 | Keeps stopwords out of topic labels |
| Topic representation | class-based TF-IDF | Unsupervised; no predefined categories |

## 6.2 Outlier Rate

Table 18: BERTopic topic assignment statistics

| Metric | Value | Note |
|---|---|---|
| Total abstracts processed | 771 | (97.2% of the 793-paper corpus had abstracts) |
| Documents assigned to topics | 605 | 78.5% |
| Outliers (Topic −1) | 166 | 21.5% |
| Number of topics | 7 | After hierarchical merging (nr_topics = 8) |
| Largest topic | 391 | Topic 1: GH treatment efficacy & adult height outcomes |

**Origin of the 21.5% outlier rate.** HDBSCAN designates documents as "outliers" (Topic −1) when they do not strongly belong to any cluster. In our corpus, the outlier rate settled at 21.5% at the baseline seed. The outliers occur because: (1) the literature spans multiple specialties (clinical endocrinology, genetics, epidemiology), creating a heterogeneous vocabulary landscape; (2) general GH treatment papers that discuss multiple topics simultaneously may not fit cleanly into any single cluster; and (3) the min_cluster_size threshold of 10 requires each topic to have at least 10 documents, relegating smaller thematic niches to the outlier pool. We additionally applied hierarchical topic merging (nr_topics = 8) to consolidate the initial HDBSCAN clusters into 7 interpretable topics; this merging step does not reassign outliers.

An outlier rate of roughly 20% is well within the typical range for BERTopic applied to specialized medical corpora of this size. Outlier documents are *not* excluded from the analysis—they are counted in the temporal evolution figures and contribute to the overall topic landscape.

## 6.3 Hyperparameter Sensitivity Analysis

To assess the robustness of the topic solution, we conducted a grid search across 20 hyperparameter configurations, varying UMAP dimensionality (3, 5, 7, 10) and HDBSCAN min_cluster_size (10, 15, 20, 25, 30). Sentence embeddings were computed once and reused across all configurations.

Table 19: BERTopic hyperparameter sensitivity grid: full results

| UMAP | min_cs | Topics | Outliers | Outlier % | Largest | Coherence | Size CV |
|---|---|---|---|---|---|---|---|
| 3 | 10 | 21 | 122 | 15.8 | 70 | 0.0392 | 0.607 |
| 3 | 15 | 16 | 157 | 20.4 | 70 | 0.0428 | 0.463 |
| 3 | 20 | 2 | 0 | 0.0 | 728 | 0.0936 | 0.888 |
| 3 | 25 | 2 | 0 | 0.0 | 728 | 0.0936 | 0.888 |
| 3 | 30 | 2 | 0 | 0.0 | 728 | 0.0936 | 0.888 |
| **5** | **10** | **23** | **166** | **21.5** | **71** | **0.0376** | **0.616** |
| 5 | 15 | 3 | 8 | 1.0 | 705 | 0.0807 | 1.254 |
| 5 | 20 | 2 | 0 | 0.0 | 728 | 0.0936 | 0.888 |
| 5 | 25 | 2 | 0 | 0.0 | 728 | 0.0936 | 0.888 |
| 5 | 30 | 2 | 0 | 0.0 | 728 | 0.0936 | 0.888 |
| 7 | 10 | 22 | 95 | 12.3 | 147 | 0.0377 | 0.974 |
| 7 | 15 | 14 | 124 | 16.1 | 147 | 0.0446 | 0.764 |
| 7 | 20 | 2 | 0 | 0.0 | 728 | 0.0936 | 0.888 |
| 7 | 25 | 2 | 0 | 0.0 | 728 | 0.0936 | 0.888 |
| 7 | 30 | 2 | 0 | 0.0 | 728 | 0.0936 | 0.888 |
| 10 | 10 | 6 | 157 | 20.4 | 461 | 0.0681 | 1.574 |
| 10 | 15 | 17 | 143 | 18.5 | 73 | 0.0422 | 0.524 |
| 10 | 20 | 2 | 0 | 0.0 | 728 | 0.0936 | 0.888 |
| 10 | 25 | 2 | 0 | 0.0 | 728 | 0.0936 | 0.888 |
| 10 | 30 | 2 | 0 | 0.0 | 728 | 0.0936 | 0.888 |

UMAP = UMAP n_components; min_cs = HDBSCAN min_cluster_size; Coherence = mean c-TF-IDF score of top-5 words per topic; Size CV = coefficient of variation of topic sizes. Our selected configuration highlighted in yellow. *Initial HDBSCAN produced 17 topics; hierarchical merging (nr_topics = 8) consolidated these into 7 interpretable topics without reassigning outliers. Configurations producing ≤3 topics represent collapsed solutions where HDBSCAN merged almost all documents into a few broad clusters.

**Key findings from the sensitivity analysis:**

1. **13 of 20 configurations collapsed** to ≤3 topics. These collapsed solutions are uninformative, splitting the corpus into one dominant cluster and residuals.
2. **Among the 7 meaningful configurations** (>3 topics): topic counts ranged from 6 to 23, and outlier rates from 12.3% to 21.5%.
3. **Low min_cluster_size is what keeps the solution informative.** Every configuration with min_cluster_size ≥ 20 collapsed, regardless of UMAP dimensionality, which is why the threshold was lowered from 15 to 10 for this corpus.
4. **Hierarchical merging** (nr_topics = 8) consolidates the raw HDBSCAN clusters into 7 interpretable topics without reassigning outliers.

**Conclusion:** The selected configuration (UMAP dims = 5, min_cluster_size = 10, min_samples = 3, hierarchical merging to 7 topics) sits in the informative region of the parameter space. The sensitivity analysis is plotted in S_Fig1.

## 6.4 Seed Robustness

UMAP initialisation is stochastic, so the topic solution was refitted across 10 random seeds with the reported hyperparameters fixed (min_cluster_size = 10, min_samples = 3, $n$ components = 5, `nr_topics` = 8). Sentence embeddings were computed once and reused, so only the seed varies.

Table 20: Seed robustness: topic recovery across 10 UMAP random seeds.

| Metric | Value |
|---|---|
| Seeds tested | 10 |
| Seeds recovering 7 topics | 10 (100%) |
| Collapsed solutions (≤3 topics) | 0 |
| Outlier % range | 15.0–21.9 |
| Baseline (seed 87) outlier % | 21.5 |

The 7-topic structure was recovered by **every seed tested** (10/10), with no collapsed solutions. Outlier assignment varies more than the topic structure does (15.0–21.9%), which is expected: borderline abstracts move in and out of the outlier pool as the embedding shifts, while the underlying thematic partition does not. Under the previous configuration (min_cluster_size = 15, min_samples = 5) only 64% of seeds recovered the 7-topic solution; the change to min_cluster_size = 10 and min_samples = 3 was made for exactly this reason.

## 6.5 Comparison with LDA and NMF

BERTopic was compared against latent Dirichlet allocation (LDA) and non-negative matrix factorisation (NMF) on the same 771 cleaned abstracts at $k = 7$, scored with normalised pointwise mutual information (NPMI) coherence and topic diversity. Because all three are seed-sensitive, each was refitted across 5 random seeds.

Table 21: Topic-model comparison: NPMI coherence and topic diversity (mean ± SD over 5 seeds)

| Model | NPMI coherence | Topic diversity |
|---|---|---|
| BERTopic | 0.147 ± 0.029 | 0.804 ± 0.014 |
| LDA | 0.140 ± 0.008 | 0.789 ± 0.019 |
| NMF | 0.183 ± 0.000 | 0.914 ± 0.000 |

**Interpretation:** On this corpus BERTopic's coherence is statistically indistinguishable from LDA's (paired $p = 0.605$) and slightly *below* NMF's ($p = 0.047$). **We therefore do not claim a coherence advantage for BERTopic.** It was selected on three grounds coherence does not capture: it assigns thematically ambiguous abstracts to an explicit outlier class rather than forcing them into the nearest topic; it uses transfer-learned sentence embeddings, which suit a corpus of this size and a field whose terminology shifted across three decades; and its topics were the most interpretable to the clinical authors. An earlier draft described a comparison "across 56 model configurations"; that wording overstated what was done and has been corrected here and in the manuscript.

# 7 Methodological Notes and Limitations

## 7.1 Multiple Comparisons

The keyword trend analysis tests many terms at once and **is** corrected. Chi-square tests of independence were computed for each tracked term across the three regulatory phases, giving a family of 37 tests. 17 reached nominal significance ($p < 0.05$); after Benjamini–Hochberg false-discovery-rate correction 8 remain significant, and the same 8 survive the more conservative Bonferroni correction. Only those 8 are described as significant trends in the manuscript; terms that lost significance under correction are reported as suggestive only.

The open-access comparison is a single pre-specified test and needs no correction. For citation burst detection, the Z-score threshold ($z > 2$) inherently controls the false-positive rate at approximately 2.3% per year cohort; no further correction was applied because the analysis is exploratory and descriptive rather than confirmatory, and the identified burst papers have strong face validity as known landmark publications.

## 7.2 Power Analysis

**Post-hoc power for the OA comparison:** With $n_1 = 394$ and $n_2 = 399$, the study had >99% power to detect a medium effect ($d = 0.50$) at $\alpha = 0.05$, so the unadjusted null is not a power failure. It is a specification failure: the unadjusted test is well powered to detect a difference that confounding has already cancelled. Post-hoc power computed from an observed effect size adds nothing here, and we do not report it; the informative quantity is the adjusted interval in Table 8.

## 7.3 Bibliometric-Specific Considerations

1. **Citation window bias:** Older papers have had more time to accumulate citations. Our burst detection partially controls for this by normalizing within publication year, but absolute citation counts still favor older publications. The h-index and g-index are similarly age-dependent.
2. **Self-citation:** Of the 3,476 citation links internal to the corpus, 721 (20.7%) are author self-citations, a proportion typical of specialised clinical fields. The citation concentration reported in the manuscript is therefore not a self-citation artifact: the most-cited papers are consensus statements whose citations come overwhelmingly from outside the corpus. **Scope limit:** this measures only corpus-internal links (corpus paper → corpus paper), computed on the 535 records with retrievable reference lists. Citations arriving from outside the corpus are not covered, so this is not a corpus-wide self-citation rate.
3. **OA classification accuracy:** OA status was determined from OpenAlex records, which rely on automated detection (Unpaywall). Classification errors are possible, particularly for bronze OA (publisher-hosted without explicit license) and delayed OA (embargo period). Misclassification would bias toward the null hypothesis (diluting any true OA advantage).
4. **Author disambiguation:** Author names were standardized, but some residual ambiguity may persist, particularly for common surnames. The most critical disambiguation (Hokken-Koelega: 11 variants merged) was manually verified.

### 7.4 Co-citation Analysis: Dual-Source Reference Data

Reference data were obtained from two complementary sources and merged using a union strategy.

Table 22: Reference data sources and coverage.

| Source | Docs with refs | Total entries | Unique refs |
|---|---|---|---|
| WoS (CR field) | 973 | 40,716 | — |
| OpenAlex (referenced_works) | 534 | 21,335 | — |
| **Combined (union)** | **638** | **28,886** | **12,501** |

WoS references were parsed from the CR (Cited References) field in WoS plain-text export files. OpenAlex references were retrieved via the OpenAlex API (`referenced_works` field) for papers with OpenAlex IDs. Reference identifiers (OpenAlex work URLs) were resolved to DOIs via batch API queries.

Table 23: Co-citation and bibliographic coupling network statistics.

| Metric | Co-citation | Bib. coupling |
|---|---|---|
| Nodes | 1,518 | 596 |
| Edges | 16,870 | 40,984 |
| Density | 0.015 | 0.231 |
| Communities | 38 | 7 |
| Min threshold | $\geq$3 co-citations | $\geq$2 shared refs |

Louvain community detection (python-louvain, random_state = 87) was applied to both networks. The co-citation network identified 38 communities, of which 10 contained $\geq$20 nodes. Community membership and network visualizations are presented in the main text (Figures 15–16).

### 7.5 Software

All analyses were performed using:

- **Python 3.14.3**: pandas 2.3.3, NumPy 2.3.5 (data management); SciPy 1.17.0 (Shapiro–Wilk, Mann–Whitney $U$, Kolmogorov–Smirnov, D'Agostino–Pearson); matplotlib 3.10.8 (visualization); NetworkX 3.6.1 (networks); python-louvain (community detection)
- **BERTopic 0.17.4**: sentence-transformers 5.2.2 (all-MiniLM-L6-v2), UMAP-learn 0.5.11, HDBSCAN (via scikit-learn 1.8.0)
- **R 4.4.1**: bibliometrix 5.2.1 (Bradford's Law, Lotka's Law, thematic map)
- **Bootstrap**: Custom implementation (10,000 resamples, seed = 42)
