# Supplementary material for: Three decades of recombinant human growth hormone research in children born small for gestational age: a bibliometric analysis (1996–2025)
Source: Front Pediatr. 2026 Sep 9;14:1903046. doi: 10.3389/fped.2026.1903046 (PMC13597677; doi:10.3389/fped.2026.1903046)
Supplement: Supplementary file 3 [file Image1.pdf]

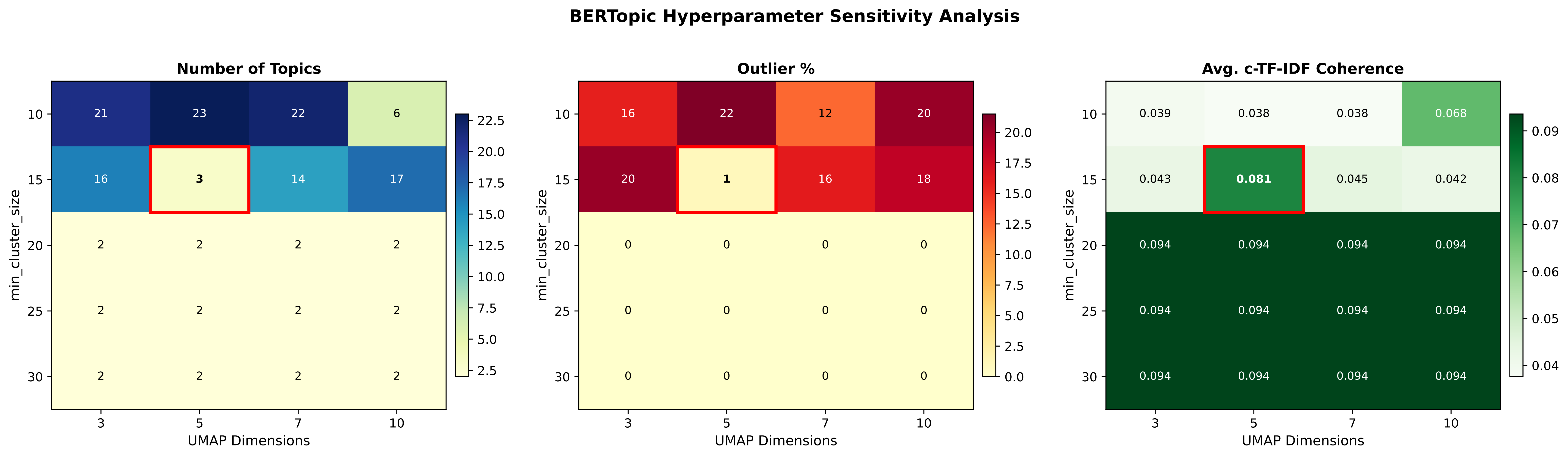
BERTopic Hyperparameter Sensitivity Analysis
Number of Topics
Outlier %
Avg. c-TF-IDF Coherence
min_cluster_size
UMAP Dimensions
10
15
20
25
30
3
5
7
10
21
23
22
6
16
3
14
17
2
16
22
12
20
20
1
16
18
0
0.039
0.038
0.038
0.068
0.043
0.081
0.045
0.042
0.094
