# Supplementary material for: Three decades of recombinant human growth hormone research in children born small for gestational age: a bibliometric analysis (1996–2025)
Source: Front Pediatr. 2026 Sep 9;14:1903046. doi: 10.3389/fped.2026.1903046 (PMC13597677; doi:10.3389/fped.2026.1903046)

Author Productivity: Lotkas Law

Inverse power law: beta = 2.25, R2 = 0.917, KS p = 0.2997

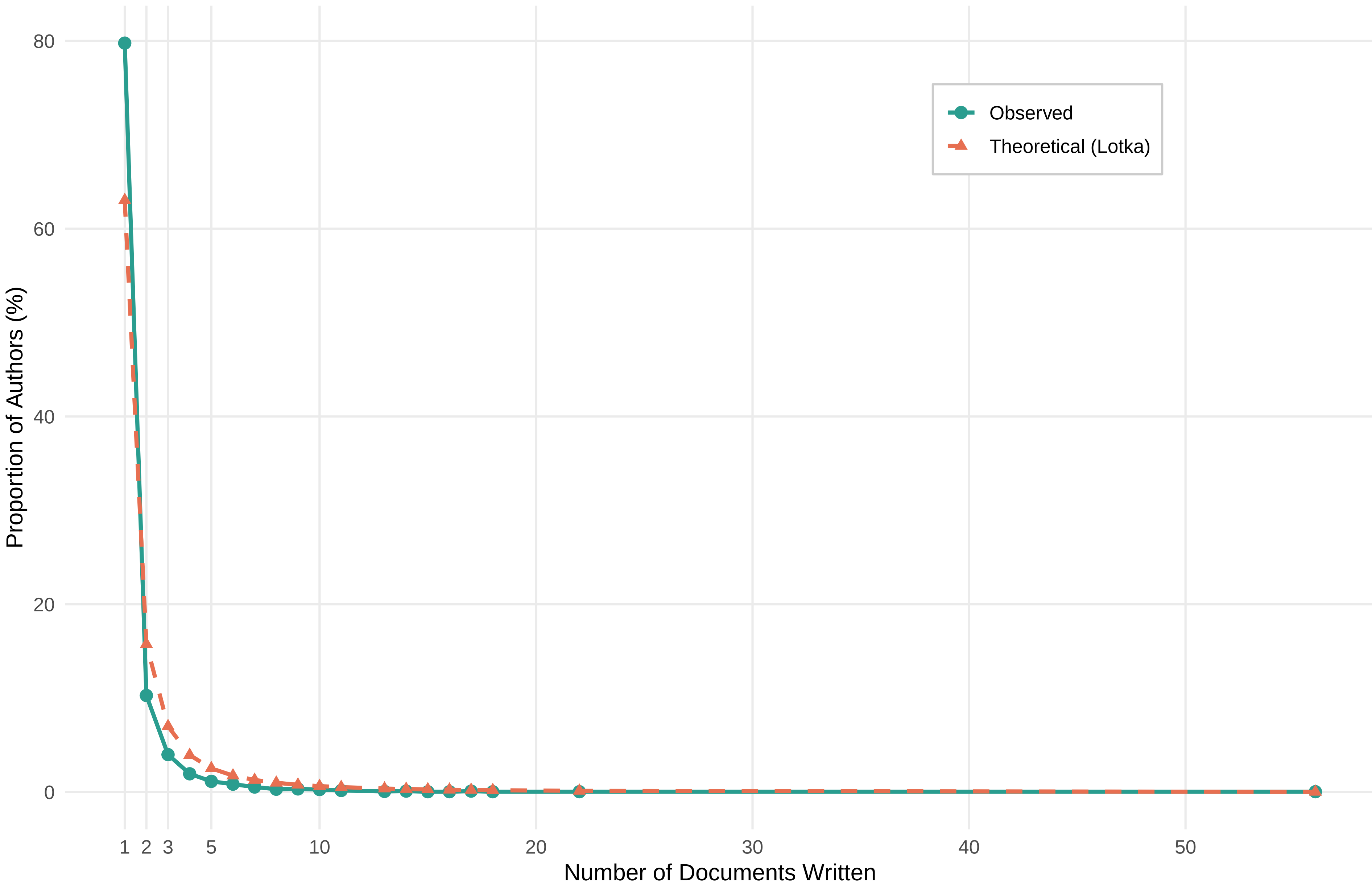

Supplement: Supplementary file 4 [file Image2.pdf]
