# Supplementary material for: Three decades of recombinant human growth hormone research in children born small for gestational age: a bibliometric analysis (1996–2025)
Source: Front Pediatr. 2026 Sep 9;14:1903046. doi: 10.3389/fped.2026.1903046 (PMC13597677; doi:10.3389/fped.2026.1903046)
Supplement: Supplementary file 5 [file Image3.pdf]

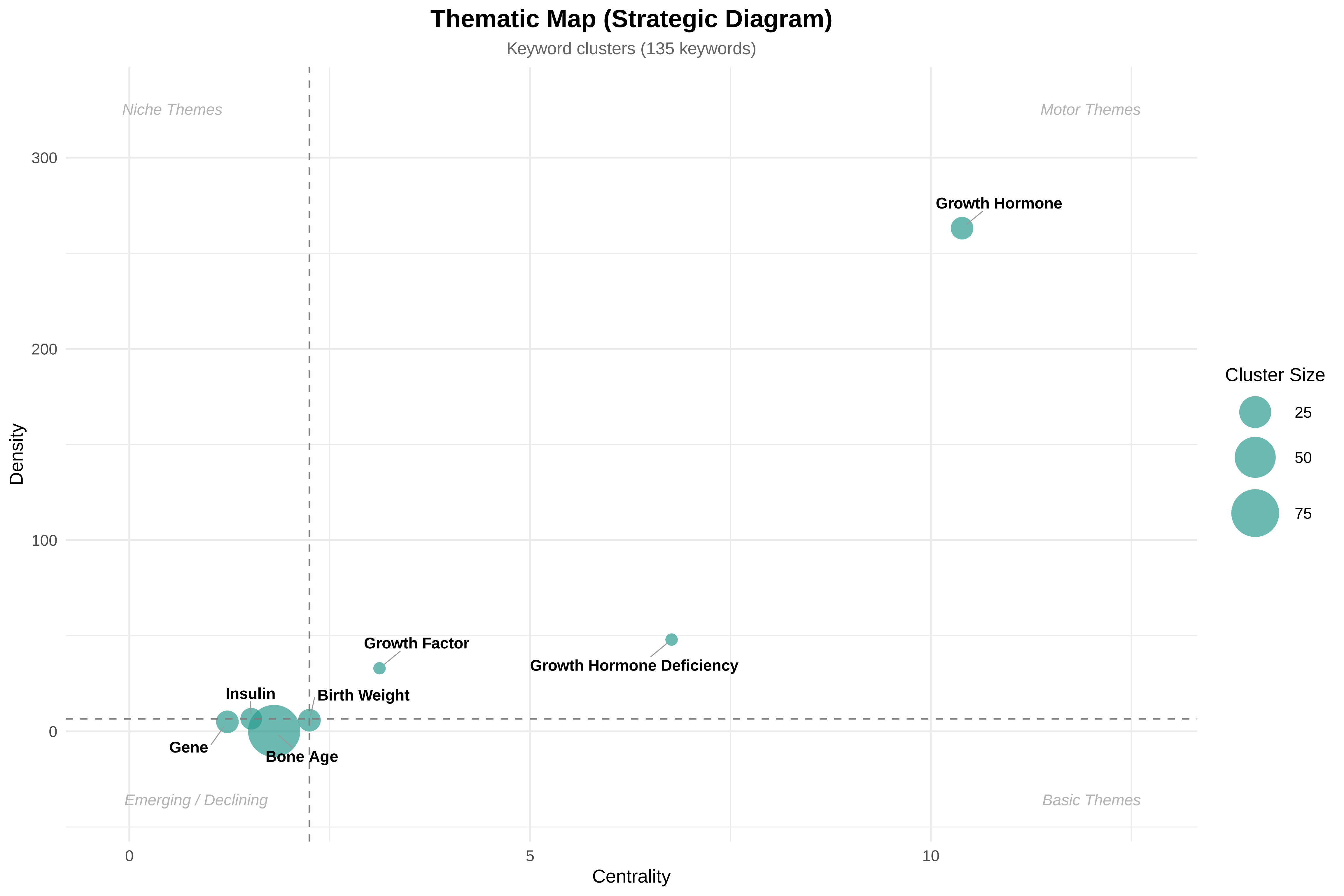

Thematic Map (Strategic Diagram)
Keyword clusters (135 keywords)
Niche Themes
Motor Themes
Emerging / Declining
Basic Themes
Growth Hormone
Growth Factor
Growth Hormone Deficiency
Insulin
Birth Weight
Gene
Bone Age
Density
Centrality
300
200
100
0
5
10
Cluster Size
25
50
75
