# Supplementary material for: Three decades of recombinant human growth hormone research in children born small for gestational age: a bibliometric analysis (1996–2025)
Source: Front Pediatr. 2026 Sep 9;14:1903046. doi: 10.3389/fped.2026.1903046 (PMC13597677; doi:10.3389/fped.2026.1903046)
Supplement: Supplementary file 6 [file Image4.pdf]

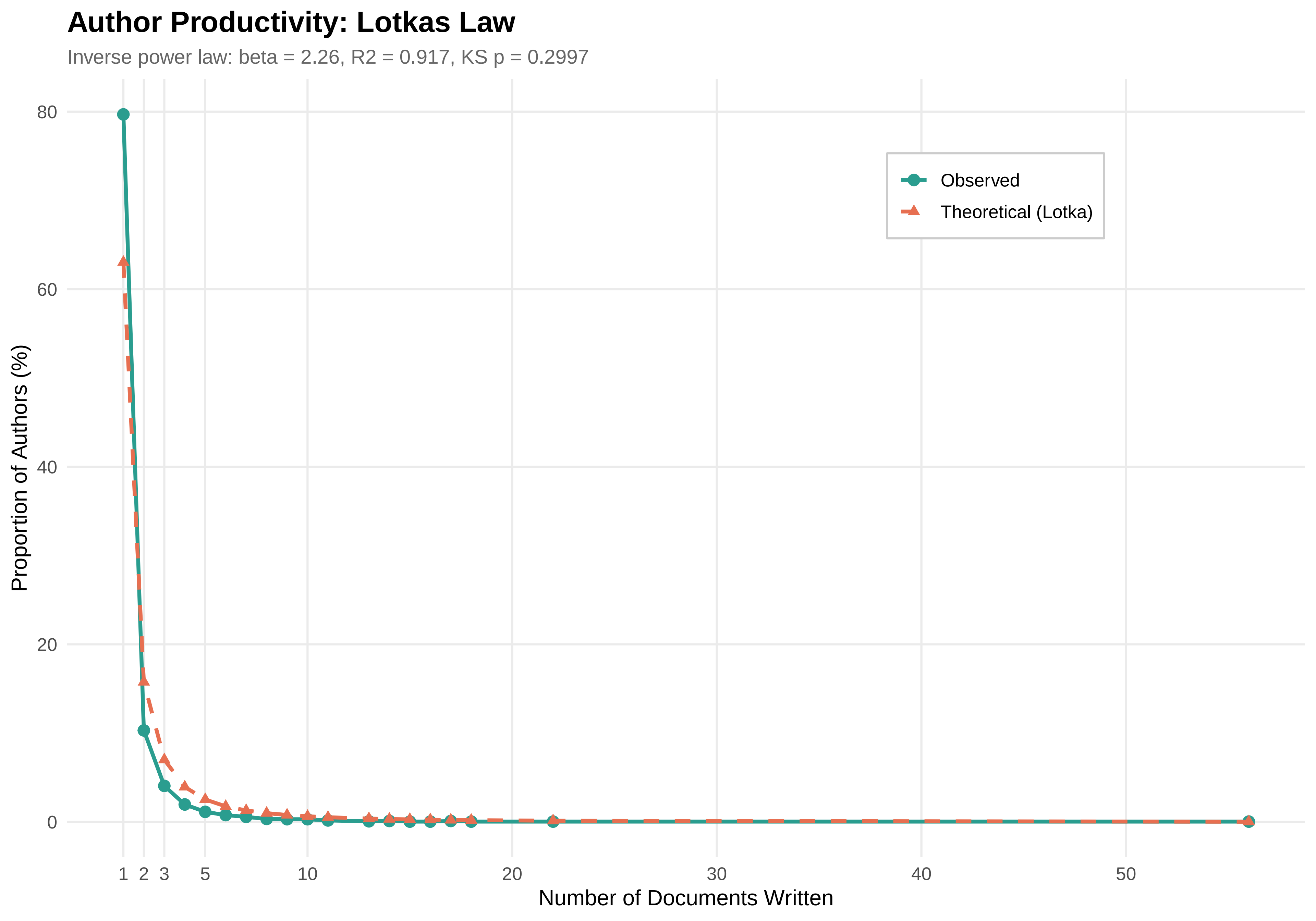

Author Productivity: Lotkas Law
Inverse power law: beta = 2.26, R2 = 0.917, KS p = 0.2997
Observed
Theoretical (Lotka)
Proportion of Authors (%)
80
60
40
20
0
1
2
3
5
10
20
30
40
50
Number of Documents Written
