# Supplementary material for: Three decades of recombinant human growth hormone research in children born small for gestational age: a bibliometric analysis (1996–2025)
Source: Front Pediatr. 2026 Sep 9;14:1903046. doi: 10.3389/fped.2026.1903046 (PMC13597677; doi:10.3389/fped.2026.1903046)
Supplement: Supplementary file 7 [file Table1.pdf]

S_Table1.

Year-by-year publication counts and mean citations per article (1996–2025)

Year	Publications	Mean cites/article	Year	Publications	Mean cites/article
1996	13	50.3	2011	30	26.6
1997	17	34.1	2012	31	24.3
1998	18	48.2	2013	24	20.4
1999	6	52.7	2014	31	16.3
2000	11	43.5	2015	22	21.2
2001	7	31.3	2016	27	35.9
2002	12	38.8	2017	33	11.2
2003	26	76.6	2018	34	15.1
2004	28	40.6	2019	32	18.2
2005	27	27.0	2020	31	13.5
2006	26	34.0	2021	44	8.6
2007	34	62.3	2022	35	8.5
2008	35	21.9	2023	37	8.1
2009	22	19.7	2024	42	2.2
2010	25	32.7	2025	33	0.5

Total: 793 publications. Citation counts reflect the February 2026 snapshot; recent years show lower means because of citation lag, not lower quality.
