# Supplementary material for: Three decades of recombinant human growth hormone research in children born small for gestational age: a bibliometric analysis (1996–2025)
Source: Front Pediatr. 2026 Sep 9;14:1903046. doi: 10.3389/fped.2026.1903046 (PMC13597677; doi:10.3389/fped.2026.1903046)
Supplement: Supplementary file 8 [file Table2.pdf]

S_Table2. Top contributing countries with single-country (SCP) and multiple-country (MCP) publication counts

Rank	Country	Publications	%	SCP	MCP	MCP ratio (%)
**1**	United States	115	11.3	47	68	59.1
**2**	Netherlands	111	10.9	72	39	35.1
**3**	Germany	95	9.3	43	52	54.7
**4**	United Kingdom	73	7.2	30	43	58.9
**5**	France	64	6.3	32	32	50.0
**6**	Sweden	55	5.4	12	43	78.2
**7**	Japan	53	5.2	41	12	22.6
**8**	Spain	43	4.2	25	18	41.9
**9**	Italy	42	4.1	21	21	50.0
**10**	Denmark	31	3.0	4	27	87.1

SCP, single-country publications; MCP, multiple-country (internationally co-authored) publications. Based on OpenAlex affiliation data available for 88.3% of the corpus.
